# Neurons and Astrocytes Elicit Brain Region Specific Transcriptional Responses to Prion Disease in the Murine CA1 and Thalamus

**DOI:** 10.3389/fnins.2022.918811

**Published:** 2022-05-16

**Authors:** Jessy A. Slota, Sarah J. Medina, Kathy L. Frost, Stephanie A. Booth

**Affiliations:** ^1^One Health Division, National Microbiology Laboratory, Public Health Agency of Canada, Winnipeg, MB, Canada; ^2^Department of Medical Microbiology and Infectious Diseases, Faculty of Health Sciences, University of Manitoba, Winnipeg, MB, Canada

**Keywords:** prion, neurodegeneration, pathophysiology, synaptic dysfunction, neuroinflammation, reactive gliosis

## Abstract

Progressive dysfunction and loss of neurons ultimately culminates in the symptoms and eventual fatality of prion disease, yet the pathways and mechanisms that lead to neuronal degeneration remain elusive. Here, we used RNAseq to profile transcriptional changes in microdissected CA1 and thalamus brain tissues from prion infected mice. Numerous transcripts were altered during clinical disease, whereas very few transcripts were reliably altered at pre-clinical time points. Prion altered transcripts were assigned to broadly defined brain cell types and we noted a strong transcriptional signature that was affiliated with reactive microglia and astrocytes. While very few neuronal transcripts were common between the CA1 and thalamus, we described transcriptional changes in both regions that were related to synaptic dysfunction. Using transcriptional profiling to compare how different neuronal populations respond during prion disease may help decipher mechanisms that lead to neuronal demise and should be investigated with greater detail.

## Introduction

Prion diseases are a group fatal neurodegenerative disorders caused by transmissible proteins termed prions. The cellular prion protein (PrP^C^) expressed at high levels in brain tissue is converted into a misfolded conformation (PrP^Sc^) that can be infectious (Prusiner, 1998). PrP^Sc^ is capable of self-template directed misfolding of endogenous PrP^C^ and this process of replication permits the spread and accumulation of prions (Mabbott, 2017). PrP^Sc^ replication is associated with pathological changes that include reactive micro- and astro-gliosis, vacuolation, and eventual synaptic loss and neuronal death. Ultimately, spongiform degeneration reflects the death of neurons and results in the neurological signs and symptoms of disease (Ritchie and Ironside, 2017). However, the mechanisms that link PrP^Sc^ deposition in the brain with these pathophysiological changes remain elusive. Uncovering the pathways that result in neuronal degeneration would inform drug design and early diagnosis.

Transcriptional profiling of brain tissue from human and animals with prion disease is an approach used extensively to identify molecular mechanisms associated with the development of prion disease. All such studies have readily identified a strong transcriptional response associated with astrocytes and microglia that take on reactive phenotypes from very early in disease, long before clinical signs and symptoms are apparent (Makarava et al., 2019; Carroll et al., 2020; Scheckel et al., 2020; Sorce et al., 2020). The transcriptional changes associated with neurons are more subtle and often not detected until late in the disease course. The heterogeneous nature of neuronal sub-populations that exist throughout the brain and the ubiquitous induction of a reactive transcriptional signature within glia contributes to this effect by masking modest or region-specific changes.

In recent years technical advances that enable transcriptional profiling from increasingly small amounts of RNA has enabled studies on targeted tissues or cell populations. They are most frequently applied to animal models that recapitulate the disease seen in human cases, such as intraperitoneal inoculation of mice. These methods include fractionation of cells, dissection, and techniques to isolate actively translated RNA by Ribo-tag profiling followed by microarray or RNAseq. Our group has employed laser capture microdissection to isolate small numbers of cells from targeted regions of mouse brain to determine transcriptional changes over the course of infection with various prions adapted to infect mice. Similarly, the reactive profile of glia are overwhelming, however, we were able to detect evidence of stress and neurotoxicity in neurons of the CA1 region during early disease (Majer et al., 2012, 2019). In the current study we used an updated methodology employing RNAseq rather than cDNA microarrays to determine transcripts with altered abundance. This has a number of advantages over hybridization technology, including improving the specificity of transcript identification and increasing the dynamic range. Given the technical challenges in sample preparation and microdissection we used a relatively low sequencing coverage depth, focusing on the detection of high-abundance transcripts.

We microdissected CA1 neurons and thalamus tissues from sections cut from the brains of mice infected with the mouse adapted RML strain of scrapie. In this model, pyramidal neurons from the CA1 region are relatively invulnerable to loss until very late in infection, whereas the thalamus shows extensive neuronal pathology, including cell death and vacuolation by midway through the incubation period (Michael et al., 2020). We identified similar transcriptional signatures from reactive microglia and pan-reactive astrocytes in tissues from both regions, although some regional specific markers were attributed to astrocytes. Neuronal gene expression from each region showed little overlap in relation to the specific genes identified, however the transcriptional signatures showed commonalities in terms of the major biological processes predicted to be affected in disease, namely dendrite morphology and synaptic dysfunction.

## Methods

### Mice

Procedures involving live animals were approved by the Animal Care Committee of the Canadian Science Center for Human and Animal Health according to guidelines set by the Canadian Council on Animal Care under animal use document (AUD) # H11-020. CD1 mice were obtained at 4–6 weeks of age before intraperitoneally inoculating with 100 μL of either 2% brain homogenate from animals infected with Rocky Mountain Laboratory Scrapie (RML) or non-infectious control animals (Mock). The animals were monitored for clinical signs of RML disease including pinched abdomen, piloerection, dull ruffled coat, ulceration, gait incoordination and weight loss of up to 20% and they reached the clinical endpoint criteria by 153–161 dpi. Mice were sacrificed at 70-dpi, 90-dpi, 130-dpi, and the endpoint of ~150-dpi by isoflurane anesthesia followed by cervical dislocation. Brains were collected from the mice and divided into front, mid and hind sections, embedded in optimal cutting temperature (OCT) medium, flash frozen in dry ice/methanol and stored at −80°C until further processing.

### Laser Capture Microdissection

Microdissection of the CA1 and thalamus brain regions was performed as described previously (Majer et al., 2012, 2019). Briefly, 8 μm coronal sections were prepared from frozen brains in OCT, placed on polyethylene-napthalate (PEN) membrane slides, and stained using the LCM staining kit (Ambion) following manufacturer's recommendations. The CA1 hippocampal region and thalamus region were microdissected with the Veritas LCM instrument (Arcturus).

### Illumina Library Preparation

Total RNA was isolated from microdissected CA1 or thalamus tissues using the RNAqueous–Micro Kit (Life Technologies Inc.). RNA concentration and quality was assessed using the Bioanalyzer RNA 6000 Pico Kit (Agilent Technologies Inc.) and samples with a RNA integrity number (RIN) > 6.0 were used for sequencing.

Sequencing libraries were prepared from total RNA using the Smart-Seq v4 Ultra Low Input RNA Kit (TakaraBio) according to manufacturer's instructions and library quality was assessed using the Bioanalyzer High Sensitivity DNA Kit (Agilent Technologies Inc.). Libraries were sequenced on a NextSeq 550 system using either the Mid-output or High-output flow cells. Libraries were sequenced to a desired depth of ~30–40 million raw read pairs per library.

### RNAseq Data Pre-processing

Raw fastq files were pre-processed using the Galaxy platform. Low quality sequencing reads were trimmed and filtered with Trimmomatic (Bolger et al., 2014) using the following parameters: sliding window trimming quality > 20, drop reads average quality <25 and drop reads length <20. Reads corresponding to rRNA were then removed by filtering out any reads that successfully mapped to mouse reference rRNA sequences using Bowtie2 (Langmead and Salzberg, 2012). Cleaned reads were aligned to the mouse GRCm38.p6 genome using HISAT2 (Kim et al., 2015) with standard settings. Aligned reads were mapped to known genes from a reference GTF file obtained from Gencode and counted using FeatureCounts (Liao et al., 2014). The raw read count files were then exported for further processing and downstream analysis using custom R scripts in RStudio.

### Read Count Normalization and Differential Expression Analysis

Raw read counts normalized using DESeq2 (Love et al., 2014) according to a negative binomial model of gene-fitted mean-dispersion estimates. Differential expression analysis was also performed using DESeq2 and unless otherwise specified, differentially expressed transcripts were defined by the following criteria: base mean read count > 15, log_2_ fold change magnitude > 0.5 and FDR corrected *p* < 0.05. Normalized abundance measurements for each transcript were obtained as log_2_ transformed read counts using the vst() function of DEseq2, and were supplied for visualizations such as principal component analysis or hierarchical clustering.

### Identifying Prion Altered Transcripts and Assigning to Brain Cell Types

Prion altered transcripts were defined as the union of all differentially expressed transcripts in the CA1 at 150 dpi, thalamus at 130 dpi and thalamus at 150 dpi, resulting in a list of 2,672 transcripts. These 2,672 mouse transcripts were mapped to corresponding human HGNC IDs using the online SYNGO ID conversion tool at https://www.syngoportal.org/convert.html#:~:text=SynGO%20%2D%20ID%20conversion%20tool,ID%20type%20supported%20by%20SynGO (Koopmans et al., 2019), with 2,372 mapping successfully. These transcripts were assigned to one of 6 brain cell types (microglia, astrocytes, vascular cells, oligodendrocytes, oligodendrocyte progenitor cells (OPCs), and neurons) according to a previously published list of enriched transcripts (McKenzie et al., 2018). The majority of prion altered transcripts (1,676/2,373) were assigned to a cell type based on this list. The remaining 660 transcripts were absent from this database and were instead assigned to one of the cell types using the resource at http://www.brainrnaseq.org/ (Zhang et al., 2014). Thirty-six transcripts were absent from both databases and were not assigned to a cell type.

### Functional Enrichment Analysis

Gene set enrichment was performed using Enrichr (Chen et al., 2013; Kuleshov et al., 2016) by supplying lists of genes that were either increased/decreased in the CA1 or thalamus. Gene set enrichment was run against the GO Biological process 2021, GO Molecular Function 2021 and GO Cellular Compartment 2021 databases, unless otherwise specified. Sematic similarity of enriched biological processes was determined using Revigo (Supek et al., 2011), which was used to summarize lists of biological process gene ontologies (with *p* < 0.05) and visualize with Treemap plots.

GSEA v4.1.0 (Subramanian et al., 2005) was used to determine enrichment of known CA1 and thalamus transcripts within each sample by supplying [log_2_ transformed read counts within each sample—mean log_2_ transformed read count across dataset] for pre-ranked gene set enrichment analysis against a database of brain region enriched genes from the Allen Brain Atlas.

### Data Visualization

Plots were produced in R using the DESeq2, ggplot2, RColorBrewer, and Treemap packages. Hierarchical clustered heatmaps were produced using ComplexHeatmap (Gu et al., 2016) package with the default Pearson distance correlation methods. Z-scores were calculated using the log_2_ transformed read counts from DESeq2 and supplied for hierarchical clustering. Protein-protein interaction networks were constructed using STRING, accessed at https://string-db.org/. Protein interaction networks were further manipulated in Cytoscape v3.9.1 by mapping corresponding log_2_ fold-change values to color for each gene.

## Results

### Evaluation of Sample Quality and Workflow for Transcriptional Analysis in Microdissected Samples

**Figure 1:**
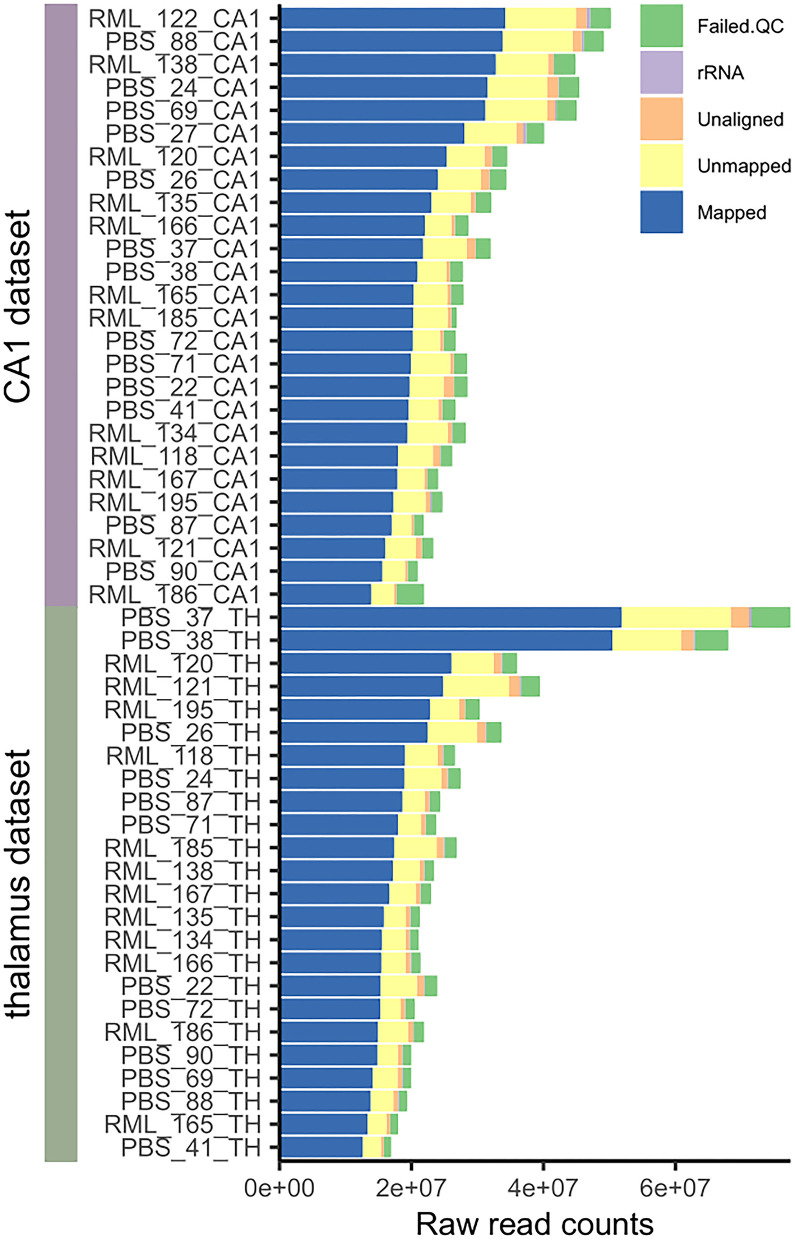
Pre-processing mapping statistics of all microdissected CA1 and thalamus samples used in the analysis. The number of raw sequencing reads that failed quality control, were removed on the basis of mapping to rRNA, were not aligned to the genome, failed to map to a valid transcript, and were successfully mapped to a transcript are indicated. We aimed for 30–40 million raw sequencing read pairs per library and successfully mapped 15–30 million read pairs per library for the analysis.

We used LCM combined with RNAseq to measure transcription in the CA1 region of the hippocampus and the thalamus of mice infected with the RML strain of scrapie, or mock-infected, at days post infection 70, 90, 130, and terminal disease (153–161 dpi). High quality sequencing data was obtained with ~60% of reads successfully mapping to known transcripts with 20–30 million reads per sample (see Figure 1). This was sufficient for the analysis of highly expressed genes, but not for an in-depth review of rare transcripts and splice variants.

**Figure 2:**
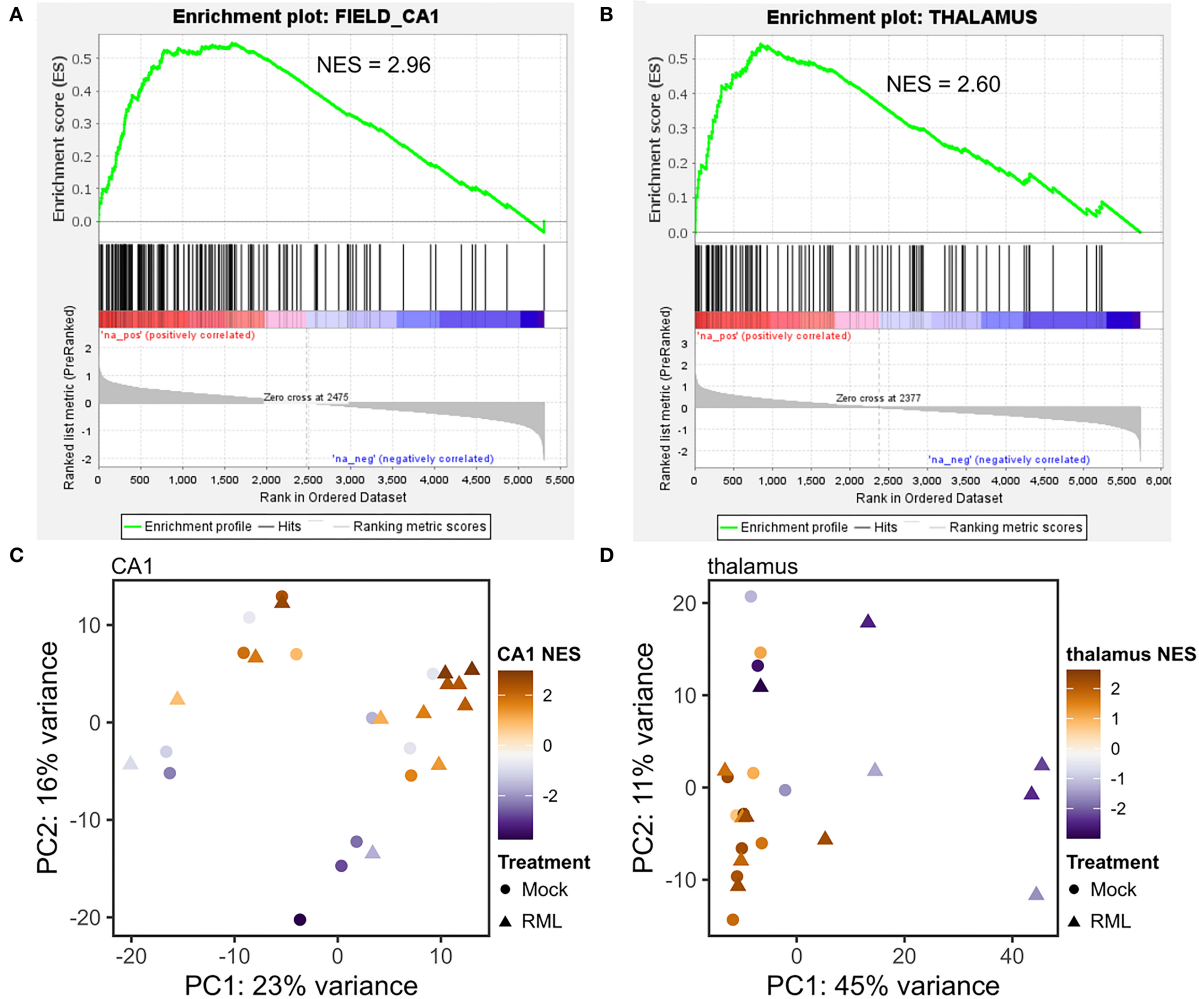
Enrichment with known gene expression profiles from the Allen brain atlas correlates with principle component analysis of microdissected CA1 and thalamus tissues. Gene expression in each microdissected sample from the CA1 or thalamus was ranked based on relative abundance and supplied to GSEA for pre-ranked gene set enrichment analysis against a database of known brain-region enriched genes obtained from the Allen brain atlas. Representative enrichment plots are shown for the CA1 **(A)** and thalamus **(B)**. Normalized enrichment scores (NES) for either the Allen brain atlas “CA1” or “thalamus” gene sets were found to correlate with principle component analysis of gene expression in microdissected tissues from the CA1 **(C)** and thalamus **(D)**.

**Figure 3:**
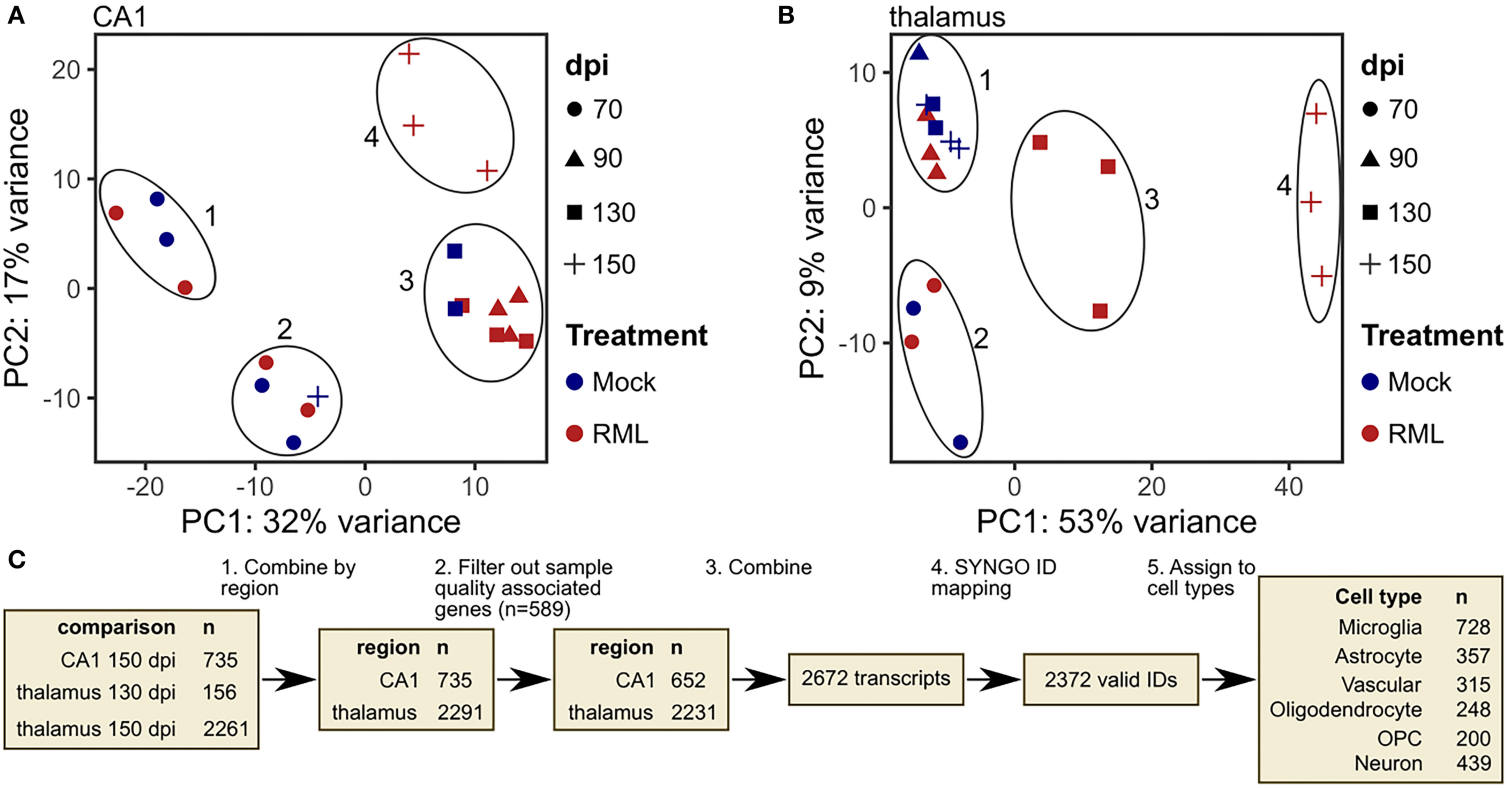
Identification of transcripts with altered abundance in microdissected CA1 and thalamus tissues from RML infected mice. **(A)** Principle component analysis (PCA) plot of microdissected CA1 tissues used for differential expression analysis with color mapped to treatment and shape indicating days post infection (dpi). **(B)** PCA plot of microdissected thalamus tissues used for differential expression analysis with color mapped to treatment and shape indicating dpi. **(C)** Schematic representation of workflow used to identify prion altered transcripts and classify them based on brain cell type.

Principle component analysis (PCA) was performed to determine the sources of variation in the data. Mock-and RML-infected transcriptomes generally clustered together at early time-points and it was not until 130-dpi in the thalamus, and terminal disease in the CA1, that variability associated with prion infection became the major correlate. Unsurprisingly, much inter-sample variation was related to the region of the brain sample rather than the disease status (see Figure 2). Stereotaxic positioning was not used when microdissecting coronal sections, so the resulting tissues could have originated from various sub-populations of CA1 pyramidal cells or different thalamic nuclei. We ranked transcripts in each sample based on relative abundance using a pre-ranked gene set enrichment analysis against a database of brain-region specific gene sets from the Allen brain atlas. The enrichment for either the CA1 or thalamus was mapped to color in the corresponding PCA plot and was found to correlate with clustering of the samples (Figures 2C,D). Samples with low enrichment scores that appeared to be outliers were removed from the datasets resulting in improved clustering based on disease status (Figures 3A,B). Samples with higher enrichment scores for either CA1 or thalamus were therefore used for differential expression analysis.

In a final assessment of variation, we examined gene expression within each dataset by grouping mock-infected samples based on PCA clustering (see Supplementary Figure 1). One group of transcripts were commonly altered in both the CA1 and thalamus (see Supplementary Figure 2). Hierarchical clustering revealed three clusters of samples from tissues that were dissected from the same mice, irrespective of disease status or brain region. We concluded that these transcriptional changes represent technical variation related to sample integrity. This cluster was enriched for genes involved in mitochondrial oxidative phosphorylation, and translation/ribosomal proteins (Supplementary Figure 2B). Another study found that similar transcripts involved in mitochondrial oxidative phosphorylation and ribosomal proteins were altered in low quality, degraded single cells (Ilicic et al., 2016)—supporting this hypothesis.

### Transcriptional Signatures of Prion Infection in the CA1 and Thalamus

RML- and mock-infected samples were grouped together based on their relatedness as determined by PCA cluster analysis and differential expression (Figures 3A,B). CA1 samples from mock- and RML-infected mice within each of the 4 clusters were used in the analysis (cluster 1 and 2–70 dpi, cluster 3–90 dpi and 130 dpi, cluster 4–RML samples from 150 dpi were compared with all mock–infected samples in clusters 1, 2, and 3). This same approach was used in the thalamus (cluster 1–90 dpi, cluster 2–70 dpi, cluster 3–RML samples at 130 dpi were compared with mock samples in clusters 1 and 2, cluster 4–RML samples at 150 dpi were compared with mock samples in cluster 1). Differentially expressed genes were defined using the following parameters: base mean read count > 15, log_2_ fold change magnitude > 0.5 and FDR-corrected *p* < 0.05. The number of differentially expressed genes identified at each comparison are summarized in Supplementary Figure 3A. We also examined the overlap of transcripts with increased and decreased abundance between each comparison in Supplementary Figures 3B,C. From this we can see significant overlap between transcripts with increased abundance in the CA1 at 150 dpi, thalamus at 150 dpi, and thalamus at 130 dpi, and conversely relatively little overlap of transcripts with decreased abundance across any of the comparisons. To determine the most robust RML-associated signature across all tissues we defined “prion altered transcripts” as the union of all differentially expressed transcripts identified in CA1-150 dpi, thalamus-130-dpi and−150-dpi samples (Figure 3C).

Differentially expressed transcripts identified at earlier time-points post infection, prior to the development of clinical signs in the mice (CA1 at 70-, 90-, and 130-dpi and thalamus at 70- and 90-dpi) did not show a clear pattern of consistent altered abundance following hierarchical clustering (Supplementary Figure 4A). We concluded that biological and/or technical variation was significant in these samples making it difficult to discern broadly conserved changes specific to prion replication. In addition, at earlier time-points prion replication is less widespread, likely affecting only a sub-population of the cells sampled, thus diluting the disease-related transcriptome. Therefore, we did not include these in our list of “prion altered transcripts.” Nevertheless, a small number of genes important for semaphorin signaling, axon extension, and chemotaxis were broadly enriched subgroups (Supplementary Figure 4B).

Prion altered transcripts were disaggregated with reference to a list of cell-type enriched genes described by McKenzie et al. to produce a specific transcriptional response associated with one of six broadly defined cell-types (McKenzie et al., 2018). These are microglia, astrocytes, vascular cells, oligodendrocyte, oligodendrocyte progenitor cells (OPCs) and neurons. Transcripts that were absent from this list were instead assigned to a cell type using the resource at http://www.brainrnaseq.org/ (Zhang et al., 2014; Figure 3C). These annotations based on cell type consensus signatures may not fully reflect the expression of individual transcripts between cell types, however, it does provide a tool to facilitate a broad functional interpretation of the data and to predict the interplay between cells within the sampled tissue.

**Figure 4:**
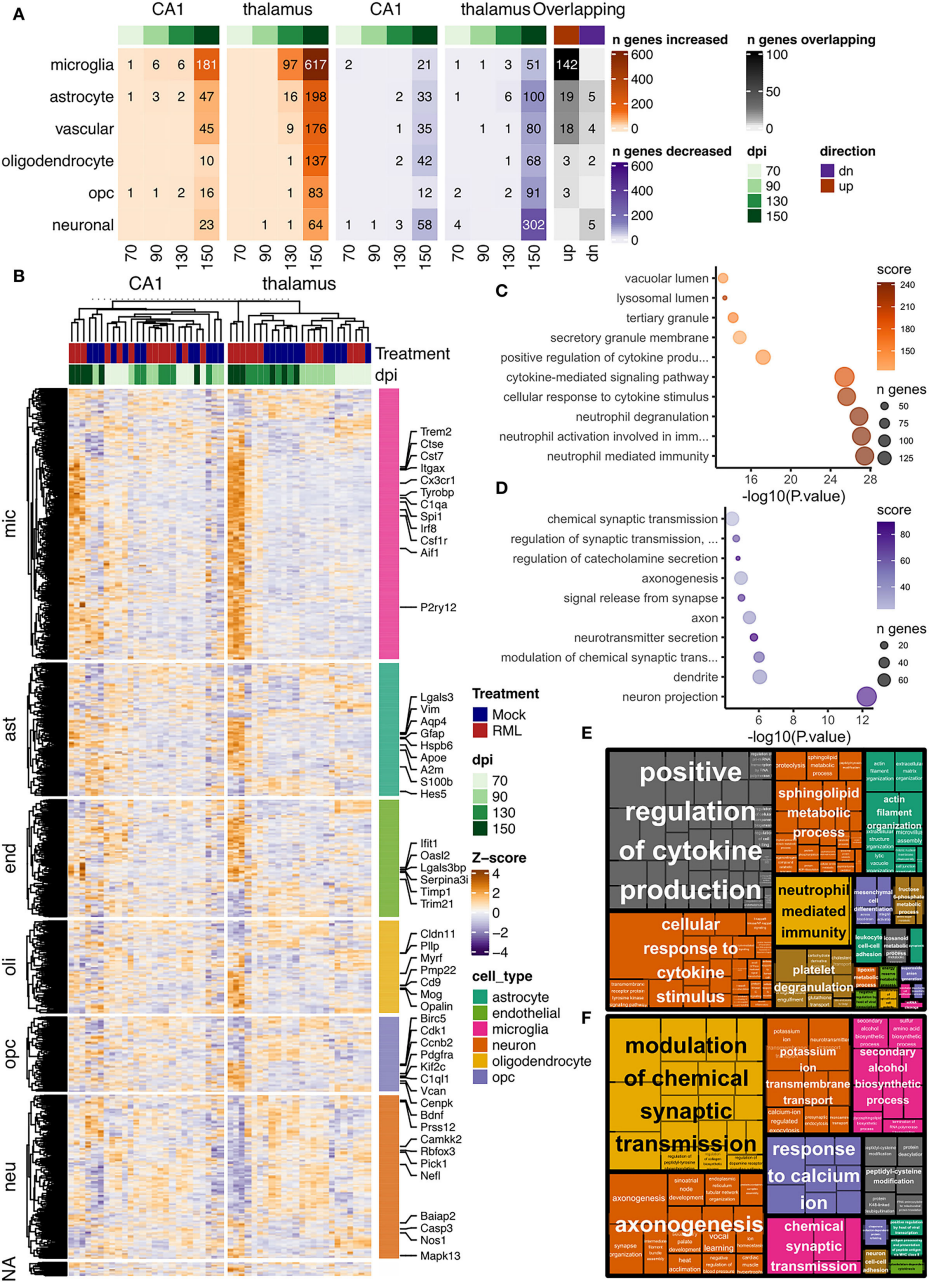
Transcriptional profiles of prion infection in the CA1 and thalamus of RML infected mice. Prion altered transcripts were defined by baseMean read count > 15, log_2_ fold change magnitude > 0.5 and FDR adjusted *p* < 0.05 in the CA1 at 150 dpi, and thalamus at 130 and 150 dpi. Transcripts were assigned to one of six broadly defined cell types with reference to a previously published list. **(A)** Number of prion altered transcripts assigned to each cell type is shown as a heatmap for those that were either increased or decreased at days post infection (dpi) −70, −90, −130, and −150. The number of overlapping prion altered transcripts in the CA1 and thalamus is also shown. **(B)** Hierarchical clustered heatmap showing relative abundance as z-scores for all prion altered transcripts in the CA1 and thalamus. Z-scores were calculated from log_2_ transformed normalized read counts output by DESeq2. Enrichr was used to identify enriched gene ontologies, and the top 10 gene ontologies that were enriched with all **(C)** increased and **(D)** decreased prion altered transcripts were plotted. Treemaps were used to visualize semantic similarity of biological process that were enriched with all **(E)** increased and **(F)** decreased prion altered transcripts.

The number of prion altered transcripts affiliated with each cell type that reached differential abundance criteria at each timepoint are provided in Figure 4A. Hierarchical clustering of all prion altered transcripts is shown in Figure 4B. Functional enrichment of gene ontologies for all prion altered transcripts that were either increased (Figure 4C), or decreased (Figure 4D), was determined using Enrichr and semantically related GO-biological processes are provided as Treemaps in Figures 4E,F. Transcripts with increased disease-associated abundance encoded genes that were highly enriched for cytokine signaling and phagocytosis, consistent with neuroinflammation. Transcripts with decreased abundance were enriched for genes involved in neuronal projections, synaptic signaling, and potassium channels, consistent with neurotoxicity.

### Altered Neuron Affiliated Transcripts in the CA1 During RML Infection Are Related to Synaptic Transmission

The CA1 region consists of densely packed pyramidal neuron cell bodies that are almost exclusively glutamatergic excitatory neurons. We have previously showed that the structural and cellular integrity of this region is maintained throughout the incubation period of the disease until clinical signs are severe (Majer et al., 2012, 2019). At this point loss of neuron cell bodies and significant infiltration of inflammatory microglia and activated astrocytes are apparent. In Supplementary Figures 5–7 we show evidence from transcript counts of gene markers that illustrates the relative difference in neuronal populations between the microdissected regions from CA1 and thalamus.

**Figure 5:**
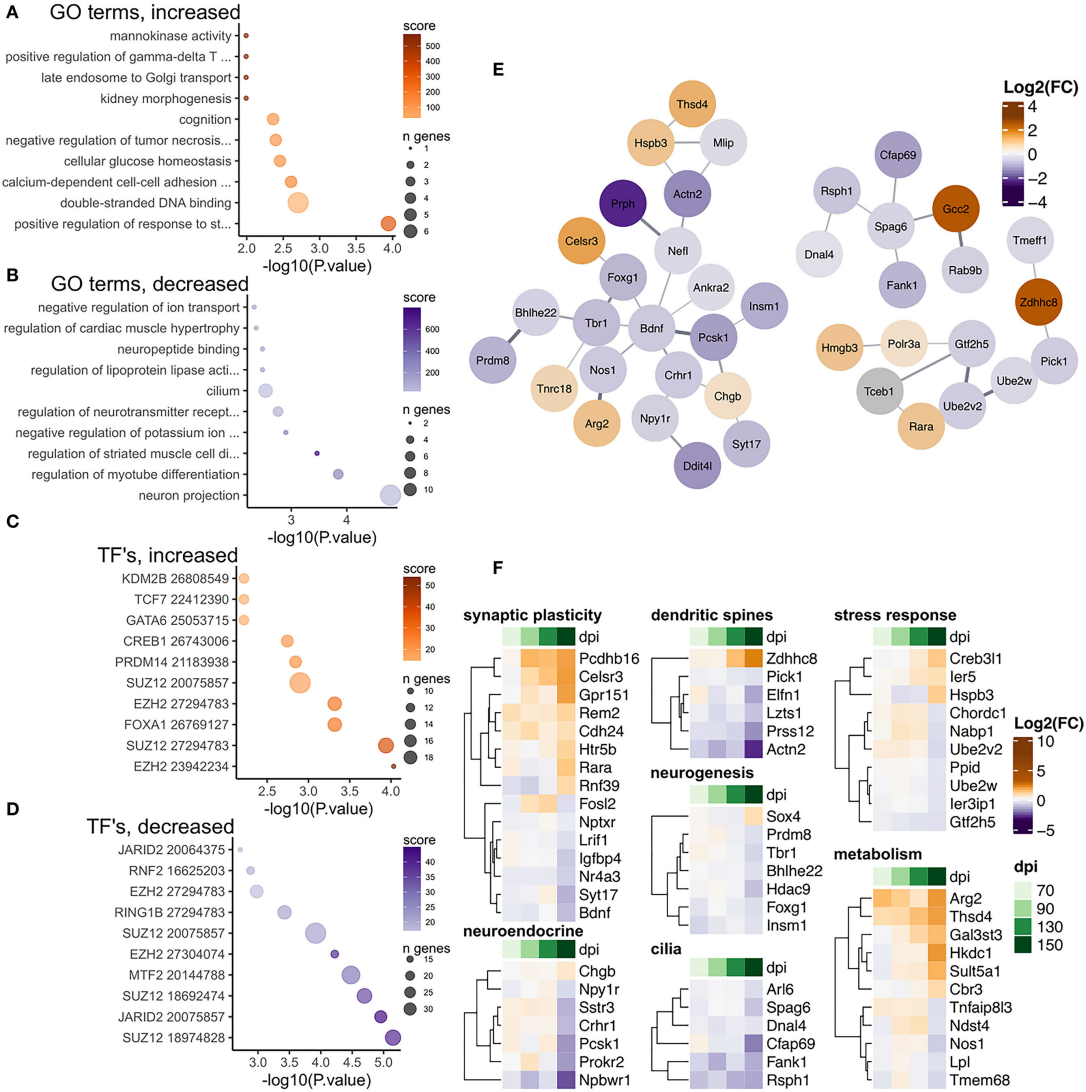
Transcriptional changes affiliated with CA1 neurons in RML infected mice at 150 dpi. Enrichr was used to identify enriched gene ontologies for CA1 neuron affiliated transcripts that were **(A)** increased and **(B)** decreased during RML disease. Enriched transcription factors were also identified for **(C)** increased and **(D)** decreased transcripts. **(E)** STRING was used to construct a protein-protein interaction network of all 123 prion altered CA1 neuron transcripts. **(F)** Heatmaps were used to visualize magnitude of change, expressed as log_2_ fold-changes at each timepoint for notable CA1 neuron transcripts that were categorized as related to synaptic plasticity, the neuroendocrine system, dendritic spines, neurogenesis, cilia, stress response, and metabolism.

Despite the relative homogeneity of the CA1 region for a neuron population enriched in glutamatergic excitatory neurons, we detected very few neuron affiliated genes with altered abundance at early time-points, and indeed at clinical endpoint (Figure 4A). We concluded from this that replication of prions does not induce a strong transcriptional profile in the soma of cells within the CA1 region. However, the processes of these cells are extensive, and our analysis doesn't include transcription and RNA processing that may occur beyond the cell bodies. In total 81 neuron genes met our criteria for altered abundance. However, with the knowledge that general neuron markers may not entirely reflect CA1 pyramidal cells specifically, we leveraged our RNAseq data to identify additional CA1 neuron markers. Criteria for neuron-altered transcripts in the CA1 was widened to include genes with a fold-change > 2 and *p* < 0.01 between Mock samples from the CA1 with those from the thalamus. In this way, 42 prion altered transcripts were “reassigned” as neuron markers specific to the CA1, increasing the number of altered neuronal transcripts to 123 (Figure 5). We further examined these markers using a publicly available mouse ISH database from the Allen brain atlas (https://mouse.brain-map.org/search/index). As an example, we compared ISH data from the Allen brain atlas with our read count data for the CA1 neuron affiliated transcripts *Lzts1, Nr4a3*, and *Rara* (Supplementary Figure 6).

There were 41/123 CA1 neuron affiliated transcripts that were increased in response to prion infection and were enriched in gene ontologies related to glucose metabolism and regulation of tissue development/neuron differentiation (Figure 5A), and the 82/123 decreased CA1 neuron transcripts were enriched in ontologies related to neuron projections, potassium transport and regulation of tissue development (Figure 5B). We used STRING to construct a protein-protein interaction network and we identified one larger network centered on *Bdnf* , *Foxg1*, and *Tbr1* (Figure 5E). Transcripts in this network encode several transcription factors known to be important regulators of neurogenesis, including *Bhlhe22* (Ross et al., 2012), *Prdm8* (Ross et al., 2012), *Tbr1* (Huang and Hsueh, 2015), *Insim1* (Monaghan et al., 2017), and *Foxg1* (Hou et al., 2020). Functional analysis of these transcripts showed many are involved in synaptic plasticity and dendritic spines assembly such as *Bdnf* (De Vincenti et al., 2019), *Actn2* (Hodges et al., 2014), *Prss12* (Mitsui et al., 2009; Levy et al., 2014), and *Pick1* (Terashima et al., 2008; Nakamura et al., 2011). *Zdhhc8* can interact with *Pick1* and it has been shown to be particularly important for regulating dendritic spine formation through acetylation of *Cdc42* (Albanesi et al., 2020). Increased abundance of the enzyme *Arg2* [reduces nitric oxide (Krystofova et al., 2018)] and decreased abundance of *Nos1* [synthesizes nitric oxide (Tricoire and Vitalis, 2012)] was also apparent. Reduction of nitric oxide induces immediate early genes important for synaptic plasticity such as c-fos, Arc, and Bdnf that show altered expression during disease (Tricoire and Vitalis, 2012). Similarly, the retinoic acid receptor *Rara* increased in abundance coincidently with the decrease of its inhibitor *Lrif1*, further implicating disruption of synaptic plasticity/LTP that is regulated by retinoic acid in the hippocampus (Nomoto et al., 2012).

We also identified transcription factors known to target these altered transcripts according to ChEA and noted that binding sites for *Suz12* and *Ezh2* were frequently enriched among transcripts with increased and decreased abundance (Figures 5C,D). Finally, we examined the fold-changes values across timepoints for notable neuron affiliated transcripts involved in synaptic plasticity, dendritic spines, cilia, metabolism, neurogenesis, neuroendocrine receptors, and stress response (Figure 5F).

### Altered Neuron Affiliated Transcripts in the Thalamus During RML Infection Are Related to Synaptic Transmission and Initiation of Cell Death

**Figure 6:**
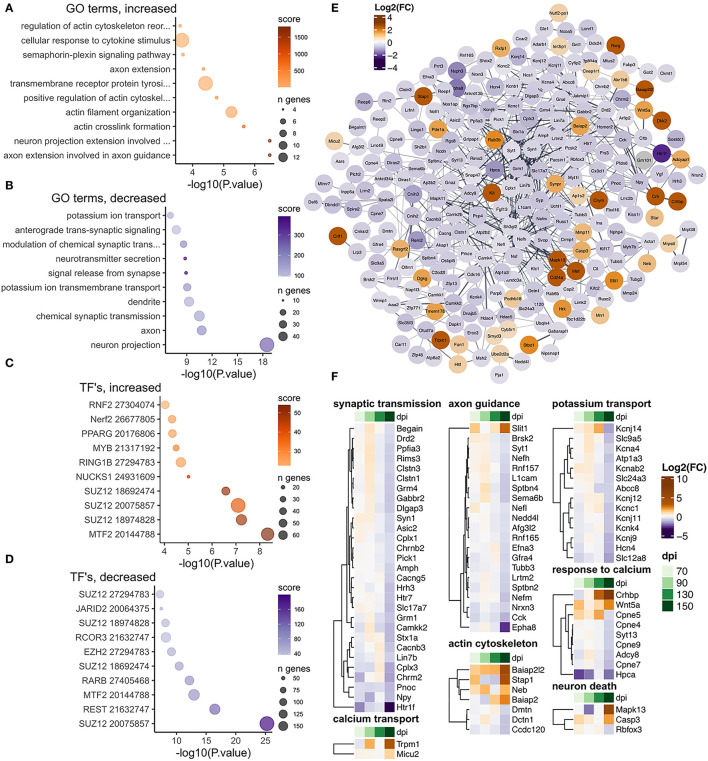
Transcriptional changes affiliated with thalamus neurons in RML infected mice at 150 dpi. Enrichr was used to identify enriched gene ontologies for thalamus neuron affiliated transcripts that were **(A)** increased and **(B)** decreased during RML disease. Enriched transcription factors were also identified for **(C)** increased and **(D)** decreased transcripts. **(E)** STRING was used to construct a protein-protein interaction network of the 366 prion altered thalamus neuron transcripts. **(F)** Heatmaps were used to visualize magnitude of change, expressed as log_2_ fold-changes at each timepoint for notable thalamus neuron transcripts that were categorized as related to synaptic transmission, calcium transport, axon guidance, actin cytoskeleton, potassium transport, response to calcium, and neuron death.

Beginning at preclinical stages of disease in RML infected mice degeneration of neurons and vacuolation is extensive in the thalamus, suggesting that this region contains cells that are particularly vulnerable to cell damage and death. For this reason, we were interested in determining whether a gene signature reflective of prion toxicity was more marked in this tissue relative to CA1 glutamatergic neurons that we found to exhibit a relatively immutable transcriptome. The thalamus sub cortical structure is less anatomically distinct and particularly heterogeneous, potentially complicating the analysis of cell-type specific transcriptomes. It contains several different nuclei and includes a variety of GABAergic, relay or interneurons and glutamatergic neurons. Glia and oligodendrocytes also make up a significant number of cells represented in the thalamus (see Supplementary Figure 5). Given this, we expected that gene signatures reflective of reactive gliosis and neuroinflammation would be evident earlier and be more extensive than in samples microdissected from the CA1, and this was indeed the case. Nonetheless, we identified signature of neuronal degeneration in the thalamus with 366 neuronal transcripts altered at endpoint RML disease (Figure 6). Interestingly, of these 89/366 were also enriched in the thalamus compared to CA1; according to criteria of a fold-change > 2 and *p* < 0.01 when comparing mock treated thalamus samples with those from the CA1. We examined these thalamus neuron affiliated transcripts using the Allen brain atlas mouse ISH database. To further illustrate the dissimilarity of neuronal markers between the CA1 and thalamus, examples of ISH data compared with our RNAseq read count data are provided for *L1cam, Cit*, and *Pcp4* in Supplementary Figure 7.

There were 64/366 neuron affiliated transcripts that were increased in the thalamus and were enriched in gene sets related to regulation of actin cytoskeleton, signaling and response to calcium ion (Figure 6A). There were 302/366 decreased thalamus neuron affiliated transcripts that were enriched in gene sets related to neuron projections, synaptic transmission, and potassium transport (Figure 6B). To examine the relationship between the proteins encoded by these transcripts, we constructed a STRING protein interaction network and noticed a high level of interconnection between altered neuron affiliated transcripts (Figure 6E). We also examined enrichment of transcription factors that are known to target these altered transcripts according to ChEA and noted that *Suz12, Ezh2*, and *Mtf2* were frequently enriched among transcripts with increased and decreased abundance (Figures 6C,D). Finally, we examined the fold-changes values across timepoints for notable neuron affiliated transcripts involved in synaptic transmission, actin cytoskeleton, neuron death, potassium transport, axon guidance, response to calcium and calcium transport (Figure 6F).

A cluster of genes in the protein interaction network (Figure 6E) with increased abundance were important for “regulation of cell death,” including *Crlf1, Met, Wnt5a, Hrk, Cd24a, Kit, Mapk13*, and *Casp3*. *Casp3* has long been known as involved in apoptosis (D'Amelio et al., 2009), while *Mapk13* has specifically been linked to prion induced synaptotoxicity in primary neuronal cultures and is activated/phosphorylated though calcium influx (Fang et al., 2018). *Rbfox3* (AKA NeuN–a marker of mature neurons) was decreased as described previously, and it is possible that neurons in this region may have been triggered to die by apoptosis. However, expression of *Casp3* could also occur in other cell types such as glia or infiltrating blood cells. Altered abundance of transcripts involved in the response to calcium and calcium transport may imply abnormal calcium signaling. Conversely, some of these increased transcripts have also been implicated in promoting neuron regeneration and/or protecting against cell death, including *Wnt5a* (Subashini et al., 2017; Zhou et al., 2017), *Dkk2* (Ghatak et al., 2017; Devotta et al., 2018), *Crlf* (Looyenga et al., 2013), *Ecel* (Kiryu-Seo et al., 2019), and *Htlf* (Helmer et al., 2013). The presence of increased transcripts associated with neuroprotection may reflect cells at different stages of response to prion replication with an early response to protect neurons followed by induction of cell death. It is also possible that different populations of neuron types in the thalamus are differentially undergoing cell death or attempting regeneration.

### Reactive Gliosis Is a Predominant Feature of the Prion Altered Transcriptome

**Figure 7:**
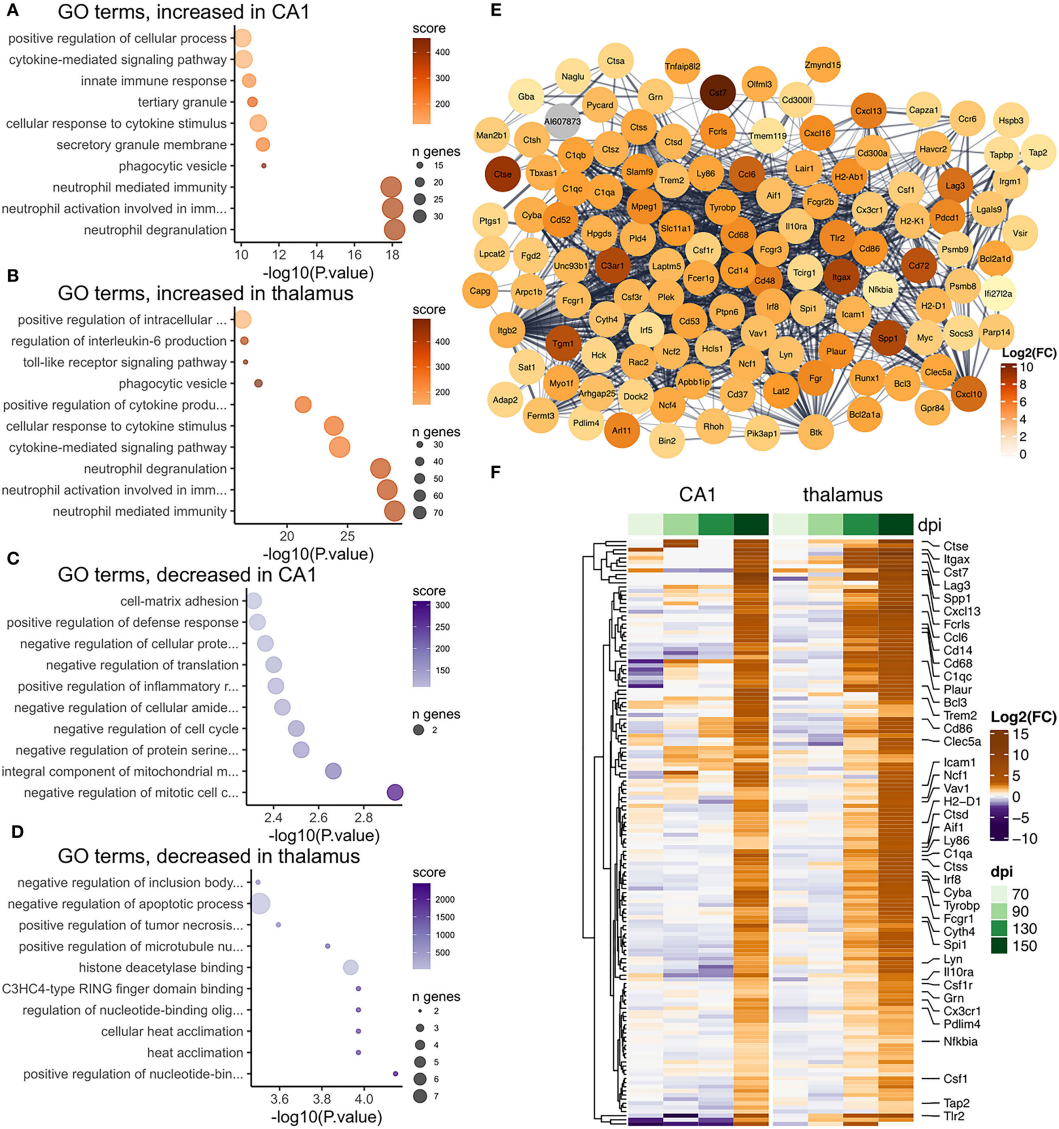
Transcriptional changes affiliated with microglia in the CA1 and thalamus of RML infected mice. Enrichr was used to identify enriched gene ontologies for microglial affiliated transcripts that were **(A)** increased in the CA1, **(B)** increased in the thalamus, **(C)** decreased in the CA1, and **(D)** decreased in the thalamus. **(E)** STRING was used to construct a protein-protein interaction network of the 142 microglia transcripts that were commonly prion altered in the CA1 and thalamus. **(F)** A heatmap was used to visualize magnitude of change, expressed as log_2_ fold-changes at each timepoint for the common microglia transcripts.

**Figure 8:**
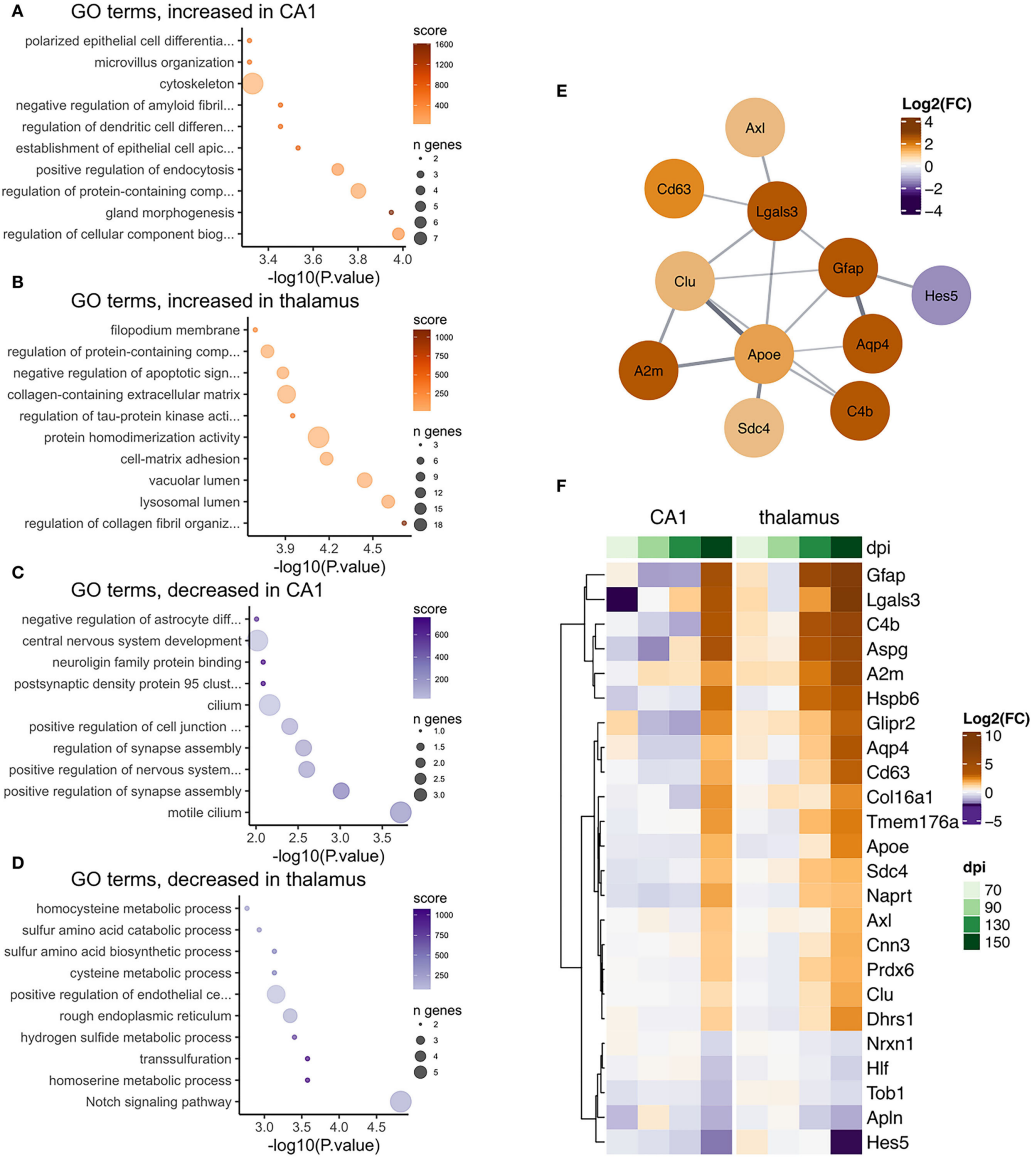
Transcriptional changes affiliated with astrocytes in the CA1 and thalamus of RML infected mice. Enrichr was used to identify enriched gene ontologies for astrocyte affiliated transcripts that were **(A)** increased in the CA1, **(B)** increased in the thalamus, **(C)** decreased in the CA1, and **(D)** decreased in the thalamus. **(E)** STRING was used to construct a protein-protein interaction network of the 24 astrocyte transcripts that were commonly prion altered in the CA1 and thalamus. **(F)** A heatmap was used to visualize magnitude of change, expressed as log_2_ fold-changes at each timepoint for the common astrocyte transcripts.

**Figure 9:**
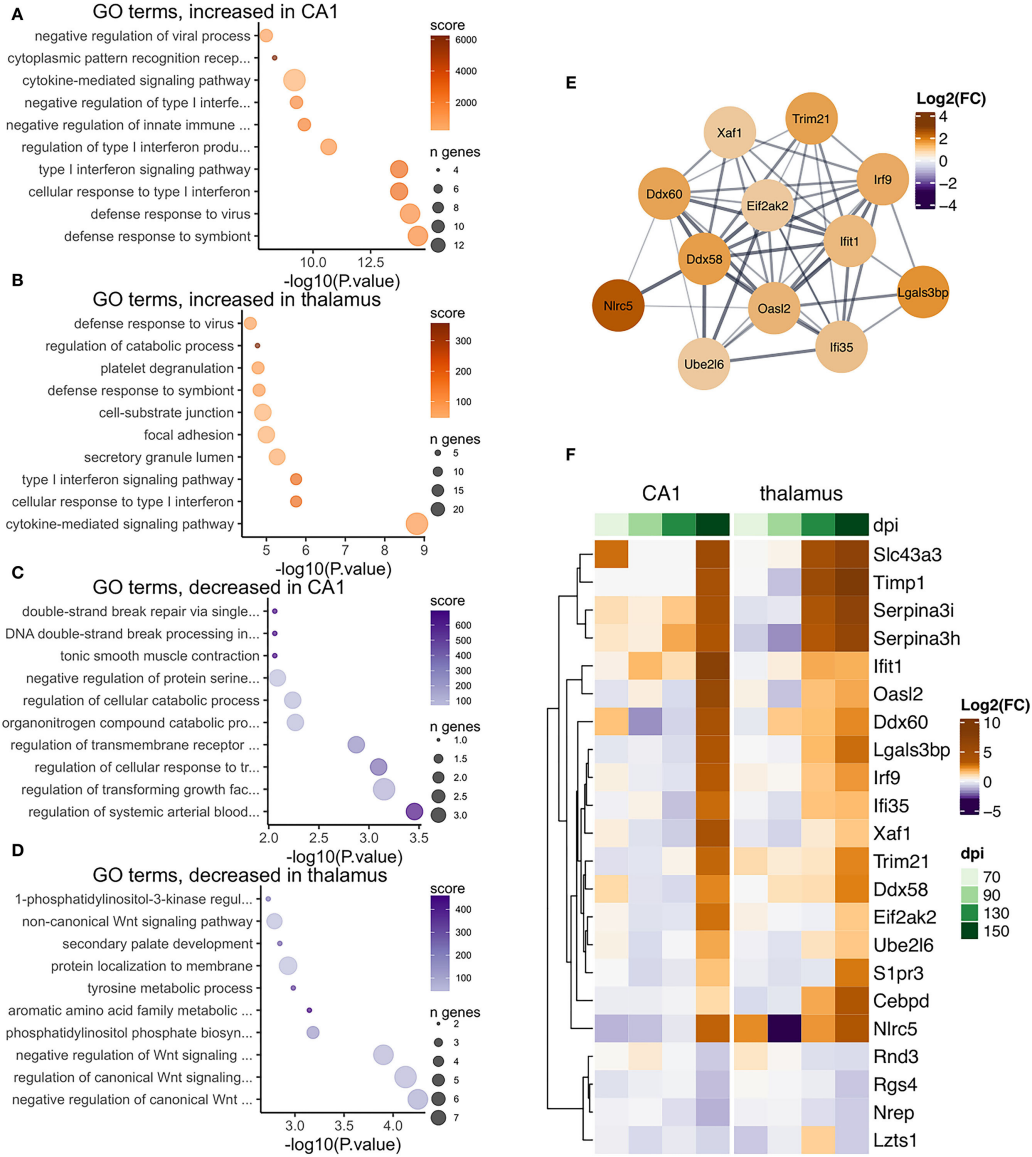
Transcriptional changes affiliated vascular cells in the CA1 and thalamus of RML infected mice. Enrichr was used to identify enriched gene ontologies for vascular affiliated transcripts that were **(A)** increased in the CA1, **(B)** increased in the thalamus, **(C)** decreased in the CA1, and **(D)** decreased in the thalamus. **(E)** STRING was used to construct a protein-protein interaction network of the 22 vascular transcripts that were commonly prion altered in the CA1 and thalamus. **(F)** A heatmap was used to visualize magnitude of change, expressed as log_2_ fold-changes at each timepoint for the common vascular transcripts.

Reactive gliosis is characterized by massive transcriptional changes, predominantly of genes involved in cell motility, phagocytosis, and the production of inflammatory mediators. Therefore, despite making up the minority of cell bodies in the microdissected CA1 tissue, the induction of high levels of glial transcripts during clinical disease is overwhelming and readily identified by-omic technologies with robust statistical significance. Expression of genes indicative of reactive gliosis was even more marked in the thalamus where the cell type's microdissected are heterogeneous. Astrocyte and microglial cell bodies are abundant within this region, and so it was unsurprising and in agreement with numerous previous studies to see transcriptional signatures of reactive gliosis from early stages of disease. To further describe the transcriptional response affiliated with reactive glia in the two regions, we examined the top 10 gene ontology terms enriched with increased and decreased prion altered transcripts in each region, performed hierarchical clustering of prion altered transcripts that were common between the thalamus and CA1, and constructed STRING protein interaction networks using the commonly altered transcripts. This analysis was performed independently using the transcripts that were affiliated with microglia (Figure 7), astrocytes (Figure 8), and vascular cells (Figure 9).

There was a high degree of overlapping microglia transcripts-−142/181 (78%, Figure 4A) were commonly increased in the CA1 and thalamus (Figure 7D). In total 617 genes annotated to microglia were increased in the thalamus, which may represent an alternative transcriptional profile or a greater degree of activation. The microglial gene expression profile in prion disease has been described in considerable detail in a number of published studies (Carroll et al., 2020; Makarava et al., 2020; Scheckel et al., 2020; Sorce et al., 2020) and our data strongly correlates with those studies. Ontological analysis shows biological processes related to cytokine signaling, phagocytosis/synapse pruning, and neutrophil mediated immunity were enriched among increased microglial transcripts in both regions (Figures 7A,B). It was striking that common prion-altered microglial transcripts were only increased in both regions (Figures 4A, 7F), some reaching very high levels (e.g., *Cst7* had a fold increase > 4,000 in the thalamus). Using STRING, many interactions were identified among the common microglial transcripts (Figure 7E), and we noted at the center of this interaction network are well-known “master regulators” of microglial activation toward a phagocytic phenotype such as *Spi1* (Zhou et al., 2019), *Irf8* (Zhou et al., 2019), *Tyrobp* (Konishi and Kiyama, 2018), *Trem2* (Konishi and Kiyama, 2018), *Aif1* (Jurga et al., 2020), and *Csf1r* (Jurga et al., 2020). No microglia transcripts were commonly decreased between the two regions, and ontological analysis suggested that decreased transcripts in each region were enriched in regulatory processes; likely related to loss of normal homeostasis function (Figures 7C,D). Altogether our results are reminiscent of a previous observation that a uniform reactive signature of prion infection can replace region-specific microglial homeostatic phenotypes (Makarava et al., 2020).

Astrocyte-associated transcripts consisted of the second highest number of altered transcripts and 24/80 (30%, Figure 4A) astrocyte transcripts were altered in both the CA1 and thalamus (Figure 8D). The 80 astrocyte genes annotated in CA1 were relatively small compared to the 298 genes identified in the thalamus. However, the limited overlap between genes in the CA1 and thalamus either suggests that the sub-population of astrocytes sampled are different in the two regions or the activation of astrocytes is context dependent. Many astrocyte affiliated transcripts were enriched in ontologies related to structural features such as collagen fibrils, cell-matrix adhesion, and filopodium in the thalamus, and cytoskeleton, microvillus organization, and cilium in the CA1 (Figures 8A–D). This was unsurprising, given that structural changes are a prominent feature of reactive astrogliosis (Schiweck et al., 2018). Furthermore, ontologies related to Notch signaling were decreased in the thalamus (Figure 8D) and the Notch-activated transcription factor *Hes5* was commonly decreased between the two regions (Figure 8F). A recent study has implicated decreased Notch/*Hes5* signaling as a mediator of astrocyte morphological changes upon inflammatory challenge (Acaz-Fonseca et al., 2019). Many of the astrocyte transcripts were enriched in ontologies terms related to redox balance, such as sulfur metabolism in the thalamus (Figure 8D). Sulfur and glutathione metabolism are important for the antioxidant capacity of astrocytes upon activation (McBean, 2017). Commonly increased in the CA1 and thalamus was the glutathione peroxidase enzyme *Prdx6*, shown to mediate protection by astrocytes in Alzheimer's disease (Pankiewicz et al., 2020). Decreased CA1 astrocyte transcripts were enriched in ontologies related to regulation of synapse assembly (Figure 8C) and these included the genes *Slitrk2* and *Nrxn1*. *Nrxn1* was commonly decreased in both regions. Neuronal *Slitrk2* regulates excitatory synapse formation (Salesse et al., 2020). *Nrxn1* is important for neuronal synaptic adhesion and neuronal differentiation (Zeng et al., 2013). Interestingly, a pre-print article suggests that *Nrxn1* is highly abundant both within neurons (including CA1 bodies) and astrocytes, and regulation of synaptic function depends on cell-type expression (Trotter et al., 2021). Finally, we supplied the 24 common astrocyte affiliated transcripts to produce a STRING interaction network (Figure 8E). Many of the interacting proteins are widely known to be induced within reactive astrocytes including *Gfap* (Hol and Pekny, 2015), *Apoe* (Fernandez et al., 2019), *Aqp4* (Ikeshima-Kataoka, 2016), and *Clu* (Foster et al., 2019). We concluded that astrocyte transcriptional changes to prion infection reflected the overall morphological and oxidative changes that accompany reactive gliosis, and at least partly depend on the different astrocyte populations found in the CA1 and thalamus.

We identified 22/80 (28%, Figure 4A) vascular transcripts that were commonly altered in the CA1 and thalamus (Figure 9D). Gene ontologies related to defense response to virus and type I interferon signaling were enriched among vascular affiliated transcripts that were increased in the CA1 and thalamus (Figures 9A,B). When we supplied the 22 common vascular transcripts to create a STRING interaction network (Figure 9E), we noted that many of the interacting proteins were related to interferon signaling such as *Ddx58, Eifak2, Ube2l6, Ifi35, Ifit1, Xaf1*, and *Irf9*. The type I interferon response is not specific to vascular cells, so it is possible these transcripts may have instead originated from glial cells. Some of the common vascular transcripts are also known as markers of reactive astrocytes, including *Lgals3bp, Timp1*, and *Serpina3h/Serpina3i* (Das et al., 2020). However, it is worth noting that a recent study found the type I interferon response to be protective in prion disease (Ishibashi et al., 2019).

## Discussion

Our main findings are that whilst sharing common microglial gene expression, different areas of the prion infected brain are associated with regional signatures related to reactive astrocytes and synaptic dysfunction of neurons. These findings align closely with our previous work, using cDNA microarrays to determine the transcriptional response to prion infection in the CA1 region of the hippocampus, as well as cerebellar granule neurons (Majer et al., 2012, 2019). Although cDNA microarrays are very sensitive for the detection of altered abundance of transcripts that bind to array “spots,” high binding stringency is not necessarily achieved for all transcripts, especially those that belong to large families of closely related transcripts and splice variants. Here we used NGS to compare transcripts with altered abundance in the same CA1 region with the thalamus of prion-infected mice, with higher gene specificity than achieved using cDNA arrays. We used a low read-depth of 20–30 million per sample to determine highly expressed genes altered in abundance in these tissues with a high degree of confidence. We also performed rigorous quality control to identify sources of technical and biological variation within the data.

Despite carefully dissecting specific populations of cells from the brains of infected mice our methods still lack resolution when discriminating transcription from the milieu of different cells within the brain. Such dissection can be imprecise as brain sections and areas chosen for dissection are not identical between mice, resulting in some sample variation. In addition, neuronal processes and other cell-cell connections are extensive in brain, more so than any other tissue. Neuronal processes contain localized “factories” of RNA processing (e.g., Bigler et al., 2017; Middleton et al., 2019; Glock et al., 2021) and translation that may provide the primary response to stresses including prion replication. This is one limitation of specifically microdissecting CA1 cell bodies in our study. We found very few RNA expression changes that could be attributed to neuronal transcription in these cell bodies, and it seems plausible that such a response could be active within neuronal processes and synapses. The thalamus also contains neurons that are known to extend to nuclei in distal regions of the brain, and so the transcriptome in this case may also be not entirely representative of neuronal activity. In addition, the diversity of both excitatory and inhibitory neurons, interneurons and glia means the transcriptional profile we detect represents an average expression of transcripts missing essential data about cell-to-cell variability of transcription.

Despite these practical limitations we used a bioinformatics approach using previously published resources (McKenzie et al., 2018) to assign transcripts to one of six broadly defined cell types—microglia, astrocytes, vascular cells, oligodendrocytes, OPCs, and neurons. Although this approach fails to account for transcripts expressed by multiple cell types, it does provide a way to de-convolute responses affiliated with different cell types. Particularly obvious was the almost overwhelming transcriptional signatures of glia, characterized by cytokine signaling, synapse pruning, neutrophil mediated immunity, regulation, and metabolism. Although this response is the major signature detected, there have been significant debate about whether the glial response is in fact a major contributor to neuron damage or if it does in fact offer protection; or merely is an accompanying response to neurodegeneration. This is an important question as modulation of glia may provide a target for drug discovery. In 2013 Gomez-Nicola et al. showed that reducing proliferation and the pro-inflammatory responses of microglia by inhibiting Csf1r slowed prion disease (Gómez-Nicola et al., 2013). Conversely, depletion of microglia has also been shown to accelerate the build-up prions in the brain and hasten the onset of clinical disease, suggesting a protective role (Zhu et al., 2016; Carroll et al., 2018). A further study awaiting publication also suggests that ablation of microglia accelerates prion disease without altering the accumulation of misfolded prion protein (Bradford et al., 2021). Given these efforts by others on studying microglia, we have concentrated our efforts on understanding the response of astrocytes and neurons to prion accumulation in the brain.

Many of the prion altered astrocyte transcripts seemed to reflect the morphological changes that accompany astrocyte activation toward a disease associated phenotype (Schiweck et al., 2018). This was also signified by decreased abundance in the CA1 and thalamus of *Hes5* and other genes involved in Notch signaling and glial cell development that can mediate such morphological changes (Acaz-Fonseca et al., 2019). We also noted that some of the astrocyte transcripts were involved in sulfur metabolism, glutathione transport, and redox. In particular, *Prdx6* was commonly increased in the CA1 and thalamus and is known to be protective in Alzheimer's disease (Pankiewicz et al., 2020). We looked in greater detail at the astrocyte transcripts that were common in the CA1 and thalamus and found many to be well-known astrocyte marker genes. Overall, the signatures resemble a pan-reactive astrocyte expression signature of chronic neurodegeneration rather than acute injury (Das et al., 2020). Of interest, we found that the proportion of the astrocyte-affiliated transcripts altered in the CA1 also altered in the thalamus was lower than that of microglia, providing some evidence that a region-specific response is triggered in astrocytes.

Some genes of interest that have been linked to potential treatments for neurodegeneration were noted. These included *Il33*, found to increase in abundance in the thalamus of prion infected mice. *Il33* has recently been found to have numerous important roles in the CNS including in acute injury and degeneration. It can be overexpressed in astrocytes from which is released to act as an alarmin released by astrocytes to induce the engulfment of synapses, shifting microglia toward a phagocytic phenotype (Gadani et al., 2015; Yang et al., 2017; Vainchtein et al., 2018). Decreased neuronal activity was recently shown to increase astrocytic *Il33* that subsequently works to regulate synaptic plasticity (Wang et al., 2021). As synapse related transcripts were dysregulated here, we speculate that *Il33* signaling may serve as a potential link between the dysfunction of neurons and astrocytes in prion disease.

Given that neuronal dysfunction leads to the clinical signs and eventual death of individuals undergoing prion disease, a major aim of this study was to identify those changes that could indicate regulatory pathways triggered by replicating prions that could be targeted by drugs. Transcripts affiliated with neurons were highly dissimilar between the CA1 and thalamus and only five transcripts were commonly decreased (*Ankrd34c, Diras2, Nefl, Nrsn2*, and *Pick1*) between the two tissues. *Diras2* is highly expressed in neurons through-out the brain, especially glutamatergic neurons, and may well be a useful marker of neuronal loss. It codes for a small Ras GTPase with as yet unknown function. Overexpression of *Diras2* in cell culture resulted in cellular vacuolation raising the possibility it may regulate cellular morphogenesis (Kontani et al., 2002). Another study knocked-down *Diras2* to identify interacting genes involved in neuronal differentiation, the regulation of cell morphology and glutamatergic signaling (Grünewald et al., 2021). *Nefl* encodes a component of the neuronal cytoskeleton and increased Nfl protein in CSF is considered to be a valuable marker of multiple neurodegenerative disorders (Bäckström et al., 2020; Ashton et al., 2021). We speculate that the decreased abundance of *Nefl* transcription observed here may be a feedback response to increased Nfl levels that are released by dying neurons. *Nrsn2* (Umschweif et al., 2021) and *Pick1* (Terashima et al., 2008; Nakamura et al., 2011) are both important regulators of synaptic signaling.

Although the transcriptomes of the CA1 cell bodies were similar between infected and control mice we did identify some gene signatures indicative of cell damage. Like our previous study using microarrays, most significant were changes in transcripts involved in synaptic plasticity and neurogenesis. Several were implicated in the structural integrity of dendritic spines whose dysfunction is the earliest pathology detected by microscopy in response to prion neurotoxicity (Fuhrmann et al., 2007). Dendritic spines extend from neuronal processes of glutamatergic neurons and are the structures on which excitatory synapses are formed. Dendritic spine loss has been directly attributed to PrP^Sc^ accumulation in primary hippocampal neuron cultures (Fang et al., 2016) and cultured cerebellar sections (Campeau et al., 2013). One hypothesis for pathology is that PrP^Sc^ oligomers induce calcium influx into neurons *via* NMDA receptors at glutamatergic synapses that results in excitotoxicity. Gene expression changes we have described in early stages of prion disease (Majer et al., 2012) as well as in functional studies from a number of groups (Torres et al., 2010; Ghirardini et al., 2020; Moon and Park, 2020) have suggested the induction of feedback mechanisms to reduce neural activity and decrease dendritic spine density as a protective mechanism to resist prion neurotoxicity. In this study we identified fewer dysregulated genes during preclinical RML infection, likely due to the reduced sensitivity of NGS with the relatively small number of samples and reads targeted in this study. We did however identify Arc and Fos, two key indicators synaptic activity identified in our previous study. Some of the prion altered transcripts we identified in affiliation with CA1 neurons in this study were *Bdnf* (De Vincenti et al., 2019), *Actn2* (Hodges et al., 2014), *Prss12* (Mitsui et al., 2009; Levy et al., 2014), and *Pick1* (Terashima et al., 2008; Nakamura et al., 2011)—all important for both dendritic spines and synaptic plasticity. Increased expression of *Arg2* and decrease in *Nos1* have also been linked to the regulation of Ca2+ influx into neurons providing further potential functional linkages between deregulated genes within CA1 neuron cell bodies. Another transcript of note that was increased was *Ceslr3*, recently reported to encode a key protein involved in synapse stability (Thakar et al., 2017). This protein has been linked to loss of synapses in Alzheimer's disease in a pre-print paper (Feng et al., 2020) and increased transcription may be a feedback response to synapse degeneration.

Given that cell death, vacuolation, and gliosis is extensive in the thalamus well-before clinical signs are apparent in this model of prion infection (Michael et al., 2020) we hoped to detect a gene signature from vulnerable to damage and death. We observed transcriptional evidence that cell death *via* apoptosis [increased *Casp3* (D'Amelio et al., 2009)] is increased in the thalamus, however, *Casp3* can also be increased in glia and infiltrating cells so it is not clear that neurons are specifically undergoing apoptosis. We identified further genes involved in synaptotoxicity *via* calcium overload such as an increase of *Mapk13* (Fang et al., 2018). Interestingly, several genes related to calcium transport and the cellular response to calcium were identified, driving functional enrichment of this pathway, similar to what we saw in CA1. These data suggest that although many of the specific transcripts identified differ between neuron populations in different brain regions, the underlying mechanisms of cell damage may be similar. Furthermore, the top transcriptional regulators identified in CA1 neurons and the thalamus had significant overlaps. Both included *Suz12, Ezh2*, and *Jarid2* that are all part of the PRC2 complex, the loss of which has been reported to lead to neurodegeneration (Peng et al., 2009; Li et al., 2013; Södersten et al., 2014; Von Schimmelmann et al., 2016).

Deciphering the mechanisms and pathways that lead to prion induced neuronal dysfunction and degeneration remains a significant challenge. Numerous studies have failed to identify a clear mechanism that links PrP^Sc^ accumulation to neuronal damage and death. Transcriptional programs are triggered; however, these may be secondary to localized disruption of distal dendrites and synapses that are not captured in complex tissues, or in microdissected neuron cell bodies. Indeed, post-transcriptional changes within neurons likely involves dysregulated mRNA transport to, or translation at, machinery in dendrites and synapses. A multitude of new technologies are evolving to examine the dynamic transcriptomes of individuals cells, which will be the next step in shedding light on how prions may be inherently neurotoxic (Keren-Shaul et al., 2017; Boisvert et al., 2018; Furlanis et al., 2019; Seweryn et al., 2020). One recently developed technique that uses a combination of translating ribosome affinity purification and ribosome profiling was recently applied to a prion mouse model, which revealed the extensive translational changes in astrocytes and microglia (Sorce et al., 2020). This study mirrored previous transcriptional data, in that few changes could be attributed to neurons. The application of these techniques will likely shed new light on the selective vulnerability of specific neuron populations and sub-populations to prions. In particular enabling the tracking of distinct cell lineages through the course of disease. However, these methods still include tissue dissociation steps that will shear neuronal processes and render the final single-cell suspensions unavoidably contaminated with closely associated material from cells such as glia. Additionally, PrP^Sc^ may well-trigger toxic cellular response distally from cell bodies that are not readily captured by transcriptional or translational alterations in single cells. Despite these limitations, the application of new—omics technologies will undoubtedly provide higher resolution data sets that will improve our understanding of the pathogenesis of prion disease. Sophisticated animal and cellular models and high-resolution microscopy for the study of protein-protein interactions will play important roles in further validation and hopefully the development of new diagnostics and drugs.

## Data Availability Statement

The datasets presented in this study can be found in online repositories. The names of the repository/repositories and accession number(s) can be found at: raw sequencing data and processed read counts have been submitted to GEO under accession # GSE201249. We have uploaded the custom R scripts for the analysis to GitHub at: https://github.com/jslota/RNAseq_CA1_thalamus_RML_infected_mice.

## Ethics Statement

The animal study was reviewed and approved by Animal Care Committee of the Canadian Science Center for Human and Animal Health, Public Health Agency of Canada, National Microbiology Laboratory, 1015 Arlington St., Winnipeg, MB, R3E 3R2, Canada.

## Author Contributions

SB: conceptualization, funding acquisition, and supervision. SB and SM: methodology. JS, SM, KF, and SB: investigation. SM and JS: data curation. JS and SB: formal analysis and writing—original draft. SB, JS, and SM: writing—review and editing. JS: figures. All authors contributed to the article and approved the submitted version.

## Funding

This work was funded by the Public Health Agency of Canada.

## Conflict of Interest

The authors declare that the research was conducted in the absence of any commercial or financial relationships that could be construed as a potential conflict of interest.

## Publisher's Note

All claims expressed in this article are solely those of the authors and do not necessarily represent those of their affiliated organizations, or those of the publisher, the editors and the reviewers. Any product that may be evaluated in this article, or claim that may be made by its manufacturer, is not guaranteed or endorsed by the publisher.
