# Supplementary material for: Neurons and Astrocytes Elicit Brain Region Specific Transcriptional Responses to Prion Disease in the Murine CA1 and Thalamus
Source: Front Neurosci. 2022 May 16;16:918811. doi: 10.3389/fnins.2022.918811 (PMC9149297; doi:10.3389/fnins.2022.918811)
Supplement: Supplementary file 3 [file Data_Sheet_3.PDF]

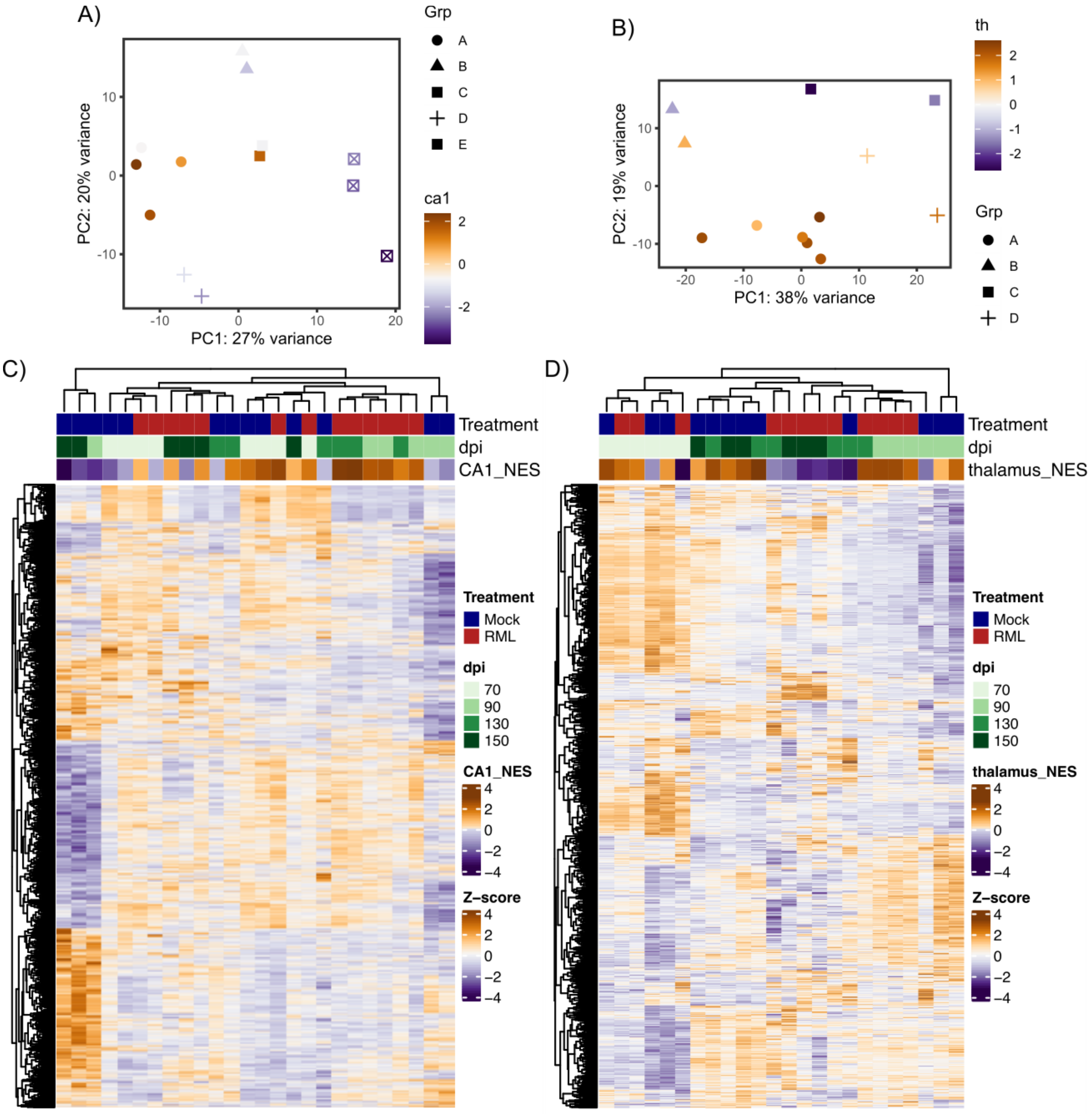


**Supplementary Figure 1. Transcripts in the CA1 and thalamus with highly variable expression between Mock infected mice. (A)** PCA plot of mock infected samples from the CA1. **(B)** PCA plot of mock infected samples from the thalamus. **(C)** Heatmap showing relative abundance of highly variable transcripts mock infected CA1 samples. **(D)** Heatmap showing relative abundance of highly variable between across mock infected thalamus samples.

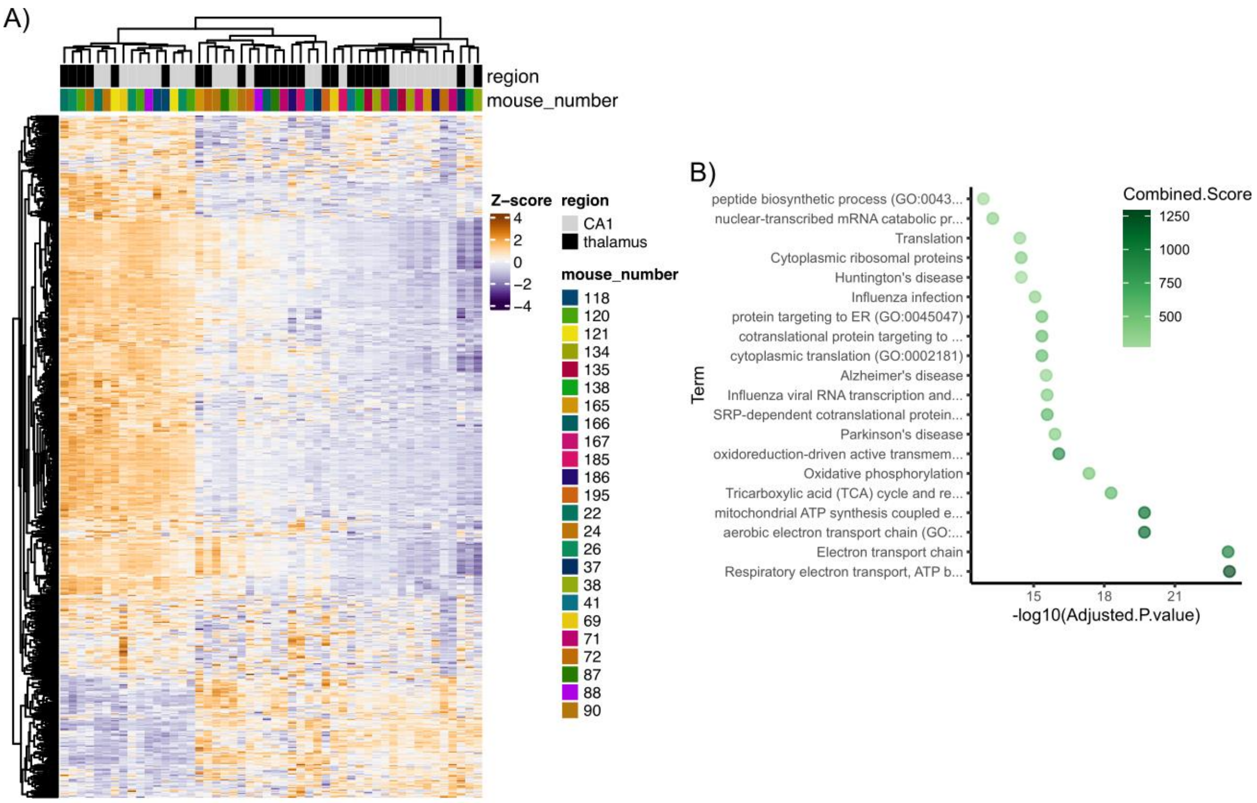


**Supplementary Figure 2. Sample quality associated genes in the CA1 and thalamus of mice. (A)** Heatmap showing relative abundance of sample-quality associated genes across samples from the CA1 and thalamus of RML and Mock infected mice. **(B)** Enriched gene sets of the sample quality associated genes.

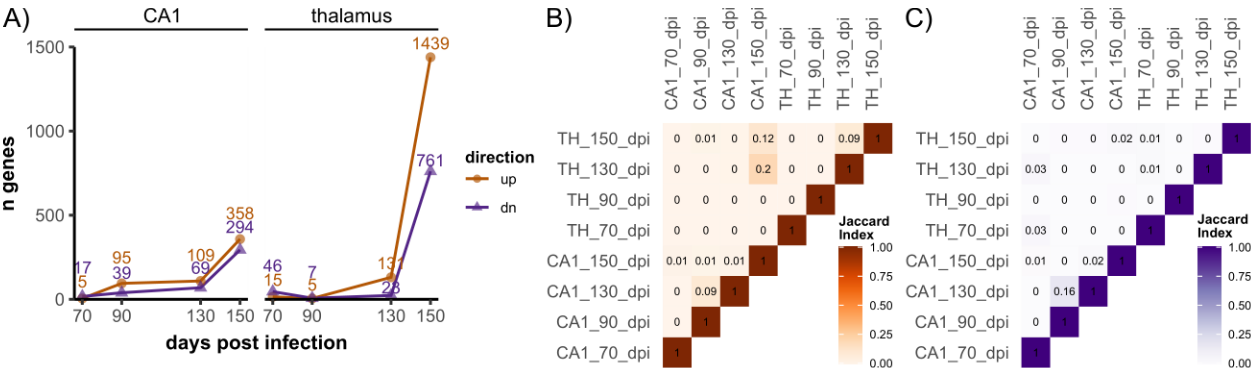


**Supplementary Figure 3. Differentially expressed transcripts in RML infected mice in the CA1 and thalamus. (A)** Number differentially expressed genes identified at each timepoint in the CA1 and thalamus. The number of transcripts with increased (up) and decreased (dn) abundance are shown separately. The number of overlapping transcripts between timepoints and tissues was calculated by Jaccard index and is shown as a separate heatmap for transcripts with increased **(B)** and decreased **(C)** abundance.

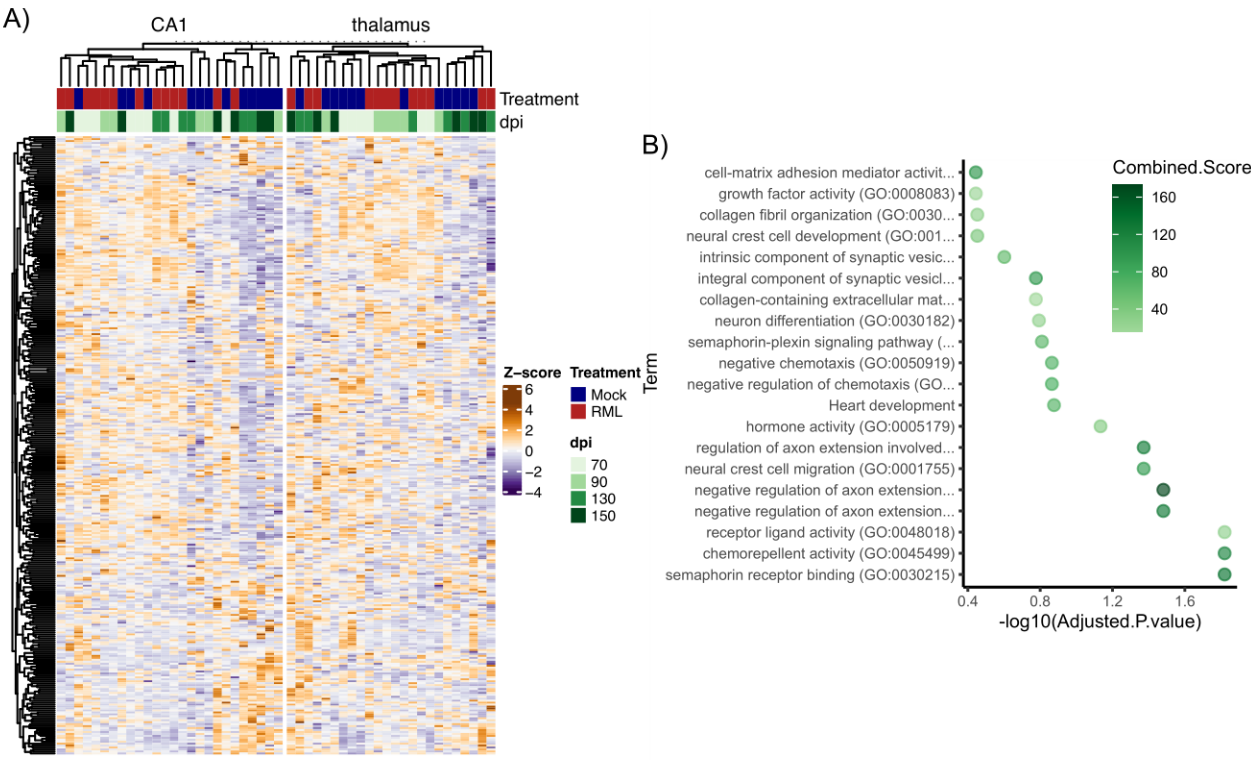


**Supplementary Figure 4. Differentially expressed genes at 70-, 90-, and 130-dpi in CA1 and 70-, and 90-dpi in the thalamus of RML infected mice. (A)** Heatmap showing relative abundance of 317 differentially expressed genes that were not considered reliably prion altered. **(B)** Enriched gene sets of differentially expressed genes.

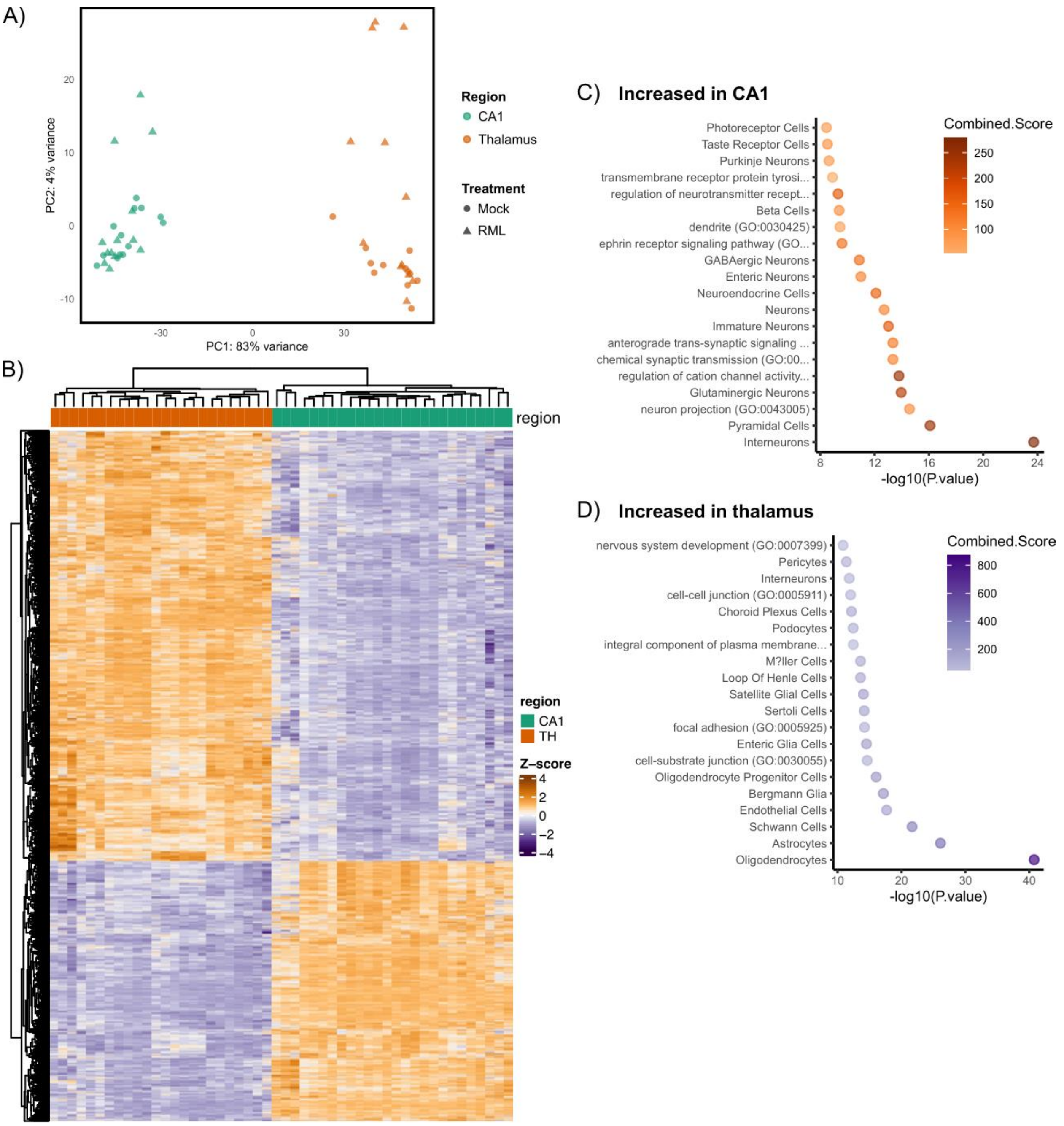


**Supplementary Figure 5. Transcriptional differences between the thalamus and CA1 after microdissection. (A)** Principal component analysis of all samples used in analysis. Samples grouped closely together based on dataset. Differentially expressed genes were identified using DESeq2 as having base mean read count > 25, FDR corrected P-value < 1x10-8 and a fold change magnitude > 2. **(B)** Hierarchical clustered heatmap showing relative abundance of 3169 differentially expressed genes across the entire dataset. Enricher was used for functional enrichment analysis against the GO Biological Process 2021, GO Cellular Component 2021, and PanglaoDB Augmented 2021 databases. **(C)** Enriched gene sets of transcripts increased in the CA1 compared to the thalamus. **(D)** Enriched gene sets of transcripts increased in the thalamus relative to the CA1.

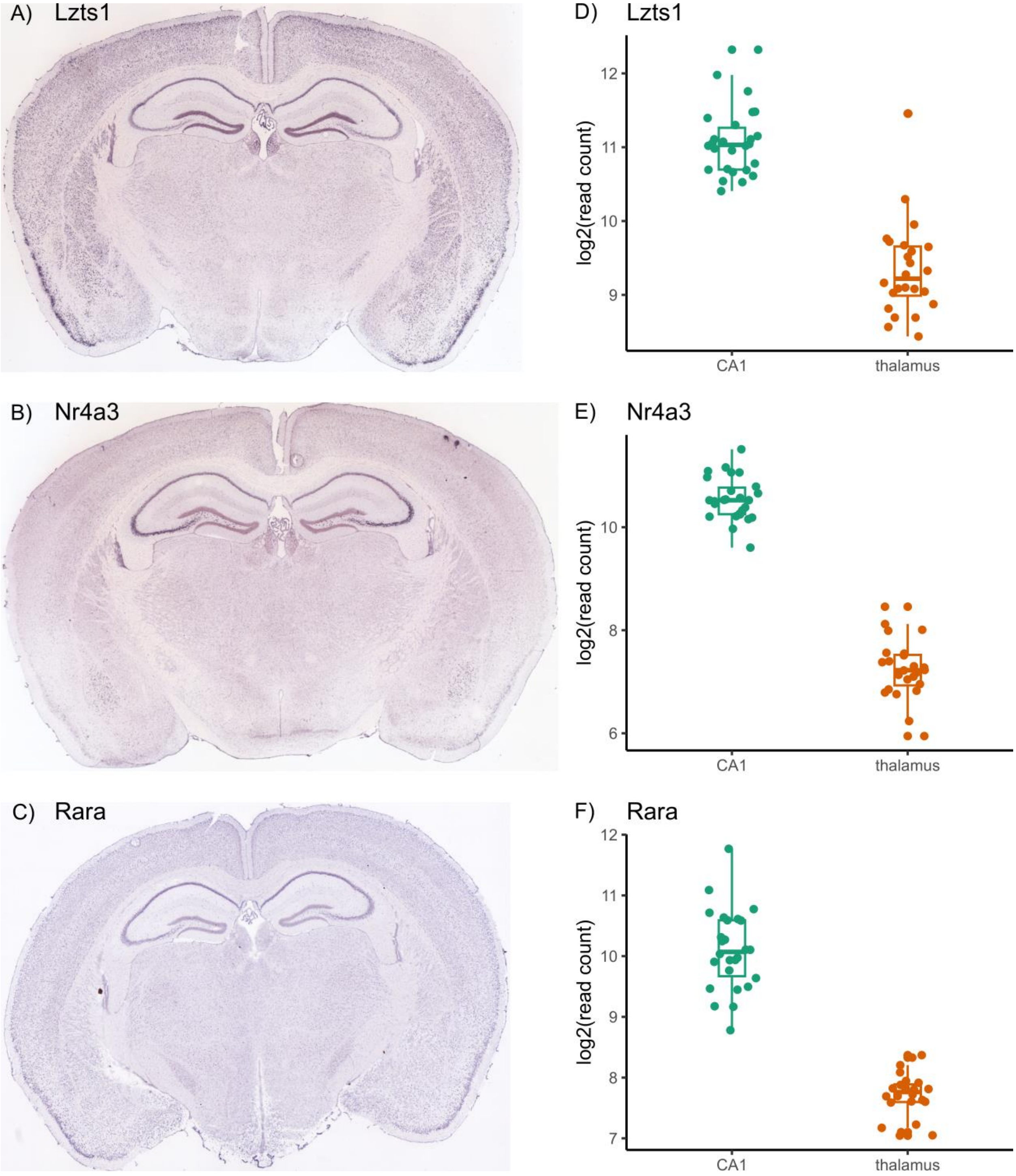


**Supplementary Figure 6. Select markers that were increased in CA1 neurons compared to thalamus neurons.** ISH images from the Allen brain atlas are shown for the markers Lzts1, Nr4a3 and Rara **(A-C)** in addition to the corresponding normalized read counts in the CA1 and thalamus **(D-F)**.

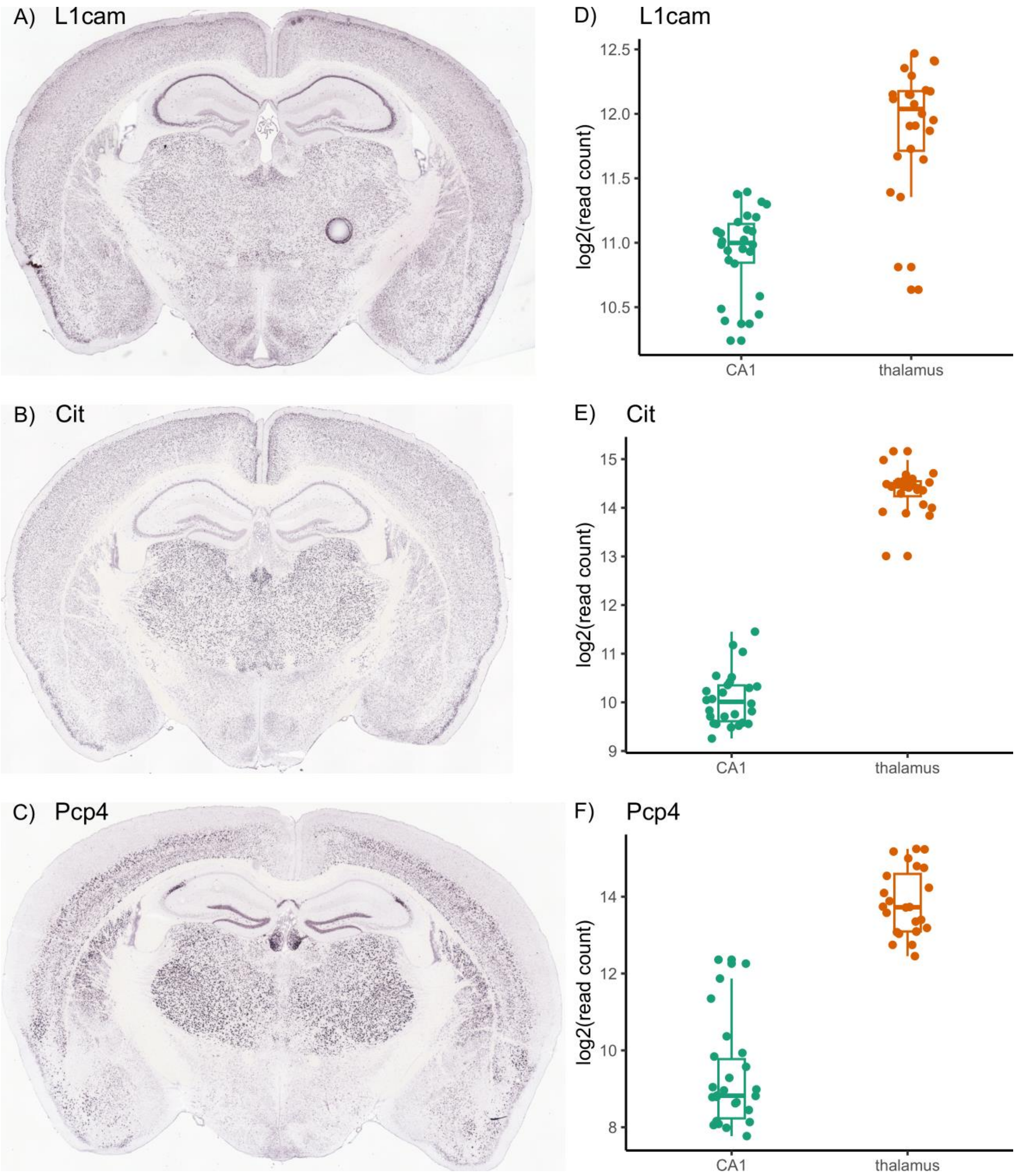


**Supplementary Figure 7. Select markers that were increased in thalamus neurons compared to CA1 neurons.** ISH images from the Allen brain atlas are shown for the markers L1cam, Cit and Pcp4 **(A-C)** in addition to the corresponding normalized read counts in the CA1 and thalamus **(D-F)**.
